# Three-Component Reaction of Benzylamines, Diethyl Phosphite and Triethyl Orthoformate: Dependence of the Reaction Course on the Structural Features of the Substrates and Reaction Conditions

**DOI:** 10.3390/molecules22030450

**Published:** 2017-03-11

**Authors:** Patrycja Miszczyk, Ilona Turowska-Tyrk, Paweł Kafarski, Ewa Chmielewska

**Affiliations:** 1 Department of Bioorganic Chemistry, Faculty of Chemistry, Wrocław University of Science and Technology, 50-370 Wrocław, Poland; patrycja.miszczyk@pwr.edu.pl (P.M.); pawel.kafarski@pwr.edu.pl (P.K.); 2 The Advanced Materials Engineering and Modelling Group, Faculty of Chemistry, Wrocław University of Science and Technology, Wybrzeże Wyspiańskiego 27, 50-370 Wrocław, Poland; ilona.turowska-tyrk@pwr.edu.pl

**Keywords:** bisphosphonates, aminophosphonates, *N*-benzylaminomethylenebisphosphonic acid, *N*-benzylaminobenzylphosphonates, synthesis of C-P bond, multicomponent reactions

## Abstract

The reaction between benzyl amines, triethyl orthoformate, and diethyl phosphite affords either bisphosphonic (compound **1**) or *N*-benzylaminobenzylphosphonic (compound **2**) acid depending on the reaction conditions. The final output of the reaction can be manipulated by the choice of reaction conditions, particularly the molar ratio of substrates.

## 1. Introduction

Bisphosphonates are a prominent class of organophosphorus compounds. They are most commonly used to treat postmenopausal- and glucocorticoid-induced osteoporosis by inhibiting the mineralization or resorption of bone [1,2,3,4]. Recently, they have also been used as drugs to treat bone problems accompanying from multiple myeloma [5,6] and as palliative agents [7,8]. Aminomethylenebisphosphonates are a subclass of bisphosphonates, and representatives from this group are promising inhibitors of physiologically important metalloenzymes, such as glutamine synthetase (a target for tuberculosis) [9], hexokinase (a promising agent against protozoan species that cause African sleeping sickness and Chagas disease) [10], undecaprenyl diphosphate synthase (a potential antibacterial agent) [11,12], HIV-1 integrase and reverse transcriptase [13,14].

**Scheme 1:**
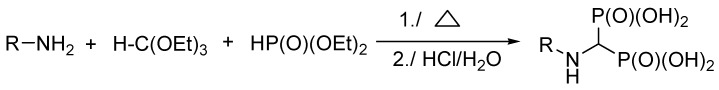
Three-component reaction of amines, triethyl orthoformate and diethyl phosphite.

Developed by Maier [15] and Kantoci et al. [16], a three-component reaction between amines, triethyl orthoformate and diethyl phosphite (Scheme 1) is perhaps the simplest and most commonly used procedure for the preparation of *N*-substituted aminomethylenebisphosphonic acids [17,18]. This reaction typically yields a complex mixture of products which are not separated but immediately hydrolysed with concentrated hydrochloric acid to provide the desired N-substituted aminomethylenebisphosphonic acids in moderate to good yields.

This reaction is also quite unpredictable and frequently affords unexpected products [17,18], and the composition of which depends on the applied conditions (molar ratio of substrates, temperature and reaction time). The complexity of this reaction could be partially explained by its mechanism. Studies using ^31^P-NMR and the isolation of all intermediates have revealed that the reaction proceeds by the formation of four intermediates which are in thermodynamic equilibrium [19].

**Scheme 2:**
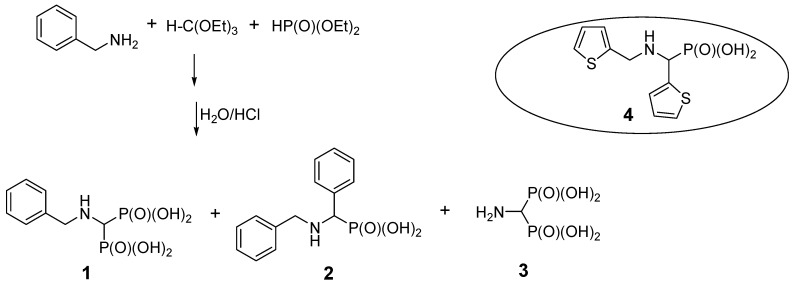
Reaction of benzylamine with triethyl orthoformate and diethyl phosphite.

When using benzylamine as a substrate, we have observed that, depending on the reaction conditions, either *N*-benzyl aminomethylenebisphosphonic acid (**1**) or *N*-benzyl aminobenzylphosphonic acid (**2**) were formed (Scheme 2). Here, we describe this reaction in some detail using structurally variable benzylamines as substrates.

## 2. Results and Discussion

### 2.1. Benzylamine as a Substrate

The products obtained in the reaction of benzylamine with triethyl orthoformate and diethyl phosphite are indicated in Scheme 2.

If the reagents were used in a 1:2:1 stoichiometric ratio (benzylamine:phosphite:orthoformate) under standard literature conditions, mixtures of the desired *N*-benzyl aminomethylenebisphosphonic acid (**1**) and *N*-benzyl aminobenzylphosphonic acid (**2**) were obtained (Table 1 entries 2 and 3). The reaction carried out at lower temperature (80 °C, entry 1) provided compound **2** as the sole product in good yield. The structure of this compound was additionally confirmed by its independent synthesis via the addition of diethyl phosphite to a Schiff base obtained from benzylamine and benzaldehyde [20]. Microwave stimulation also produced compound **2** as the sole reaction product, albeit in low yields (entries 4–7). Prolonging the reaction time to over 12 min resulted in complex mixtures of products which were difficult to separate.

It is worth mentioning that the use of 2-thiophenemethylamine as a substrate under standard conditions (1:2:1 molar ratio of substrates, 135 °C) also provided the corresponding aminophosphonic acid (compound **4**, Scheme 2) in satisfactory yield.

The formation of compounds **2** and **3** in a reaction designed to prepare the corresponding aminomethylenebisphosphonates was surprising and difficult to explain based on the known mechanism of the three-component reaction (for more detailed discussion see next paragraphs).

However, the desired bisphosphonate **1** was obtained in good yield when applying the reagents in a molar ratio of 1:4:2 (entry 8). Thus, there is the possibility to steer the course of this reaction by manipulating with ratios of substrates, which prompted us to study the behaviour of benzylamines with substituents in the aromatic ring.

In some cases, the formation of minute quantities of aminomethylenebisphosphonic acid (**3**) was also observed (this compound is clearly seen in the ^1^H-NMR spectra as a triplet at approximately 1.5 ppm with *J*_PH_ = 16.5 Hz [21]). Because this compound was previously prepared by the acid hydrolysis of *N*-benzhydrylaminomethylenebisphosphonic acid [22], we speculated that it might also be formed upon the hydrolysis of compound **1**. However, this did not appear to be the case, as prolonged refluxing of the latter in boiling concentrated hydrochloric acid failed to produce compound **3**.

### 2.2. Reactions of Benzylamines with Substituents on the Phenyl Ring

First, reactions were carried out under conditions that provided mixtures of products **5** and **6** (1:2:1 molar ratio, 135 °C, 12 h of heating). In most cases, aminophosphonates **6** were obtained as single products (Table 2, entries 3, 7–10, 14), or they predominated the reaction mixtures (entries 12, 13). Additionally, the formation of minute quantities of compound **3** was sometimes observed. However, its appearance was quite random.

**Scheme 3:**
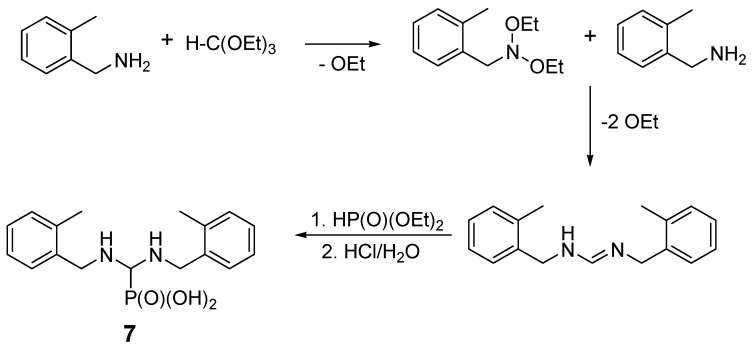
Presumed route to the formation of compound **7**.

The substituents on the phenyl ring showed significant effects when studying the series of benzylamines bearing a methyl substituent on the phenyl ring. Thus, for *m*-methylbenzylamine, aminophosphonate **6b** was obtained as the sole product of the reaction in molar ratio 1:2:1 (entry 3), whereas in the case of *p*-methylbenzylamine (in the same molar ratio of the reagents), a mixture of products was formed with bisphosphonate **5c** being the predominant one (entry 5). Lowering the temperature of the reaction to 80 °C resulted in the formation of aminophosphonate **6c** as the sole product (entry 7). Interestingly, for *o*-methylbenzyl amine (entry 1), the formation of an additional product **7** was observed. The same results and formation of products **16** were observed for *o*-chlorobenzylamine used as a substrate. This compound is the hydrolysed form of one of intermediates that was identified earlier [19]. The possible route to its formation is shown in Scheme 3. Although the combined yield of compounds **6a** and **7** was low (10%), this side product was obtained as a significant fraction, as the molar ratio of **6a**:**7** was only 73:25 (based on the ^31^P-NMR spectrum).

**Figure 1:**
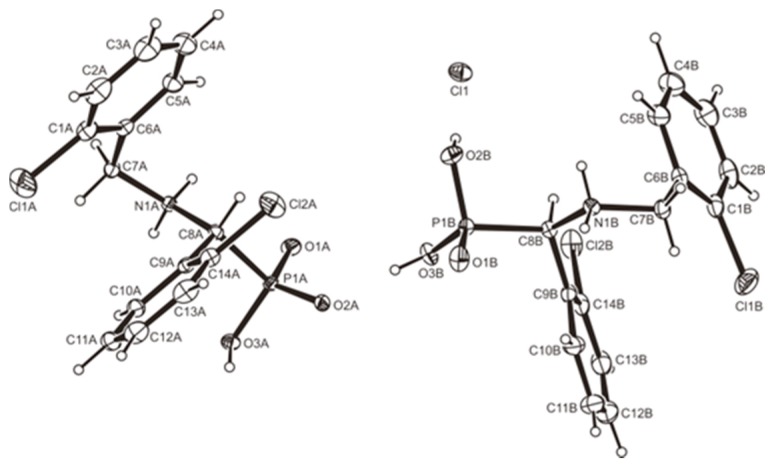
The crystal structure of molecule **6d**. Displacement ellipsoids are drawn at a 20% probability level.

Since the formation of aminophosphonic acids **6** is somewhat surprising, we succeeded in isolating one of them, compound **6d** (entry 8), in crystalline form, and thus, its structure was unequivocally confirmed by X-ray crystallography. The asymmetric unit contains two nitrogen-protonated species, one of which possess a singly deprotonated phosphonate group (thus, is zwitterionic), while the second one is fully protonated with the charge neutralized by a chloride anion (Figure 1).

In the reaction with *o*-methylbenzylamine as a substrate, the change in the molar ratio of reagents to 1:4:2 once more resulted in a change of the reaction course, and aminomethylenebisphosphonate **5a** was obtained in satisfactory yields (entry 2). The only exception was the reaction with *p*-chlorobenzylamine (entry 11), which afforded a mixture of products. Thus, steering the reaction course by manipulating the reaction conditions is also effective for other benzylamines.

**Scheme 4:**
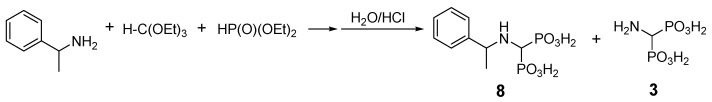
Reaction of α-methylbenzylamine.

The reaction of α-methylbenzylamine (135 °C, 1:2:1 molar ratio) gave a nearly equimolar mixture of the desired *N*-(α-methylbenzylamino)methylenebisphosphonic acid (compound **8**, Scheme 4) and aminomethylenebisphosphonic acid (compound **3**). Changing the molar ratio of the reagents to 1:4:2 resulted in the production of compound **8** as the sole reaction product.

**Scheme 5:**
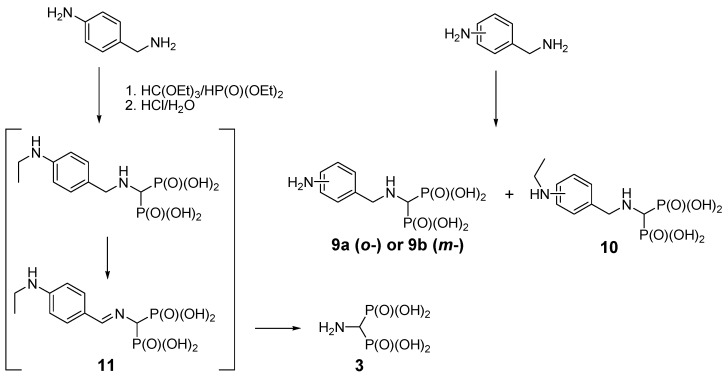
Reaction with the aminobenzylamines as substrates.

Different courses for the reaction were obtained when using aminobenzylamines as substrates. Aminomethylenebisphosphonic acid (compound **3**) was the major product in the reaction (85% yield) of *p*-aminobenzylamine. The formation of this product may be explained by the formation of the intermediate imine **11** (suggested by mass spectrometry of the crude reaction mixture), which decomposes to compound **3** (Scheme 5). As deduced from the ^1^H-NMR spectra of the crude products, the reactions of *o*- and *m*-aminobenzylamines provided complex mixtures of compounds with the expected bisphosphonates **9** and their *N*-ethylated derivatives **10** (Scheme 5) It is worth mentioning that ethylation has been previously found as an infrequent side reaction accompanying the studied three-component reaction [18,19,23]. The expected bisphosphonates **9** were isolated by crystallization from crude reaction mixture.

### 2.3. Presumable Mechanism of Reaction Leading to N-Benzyl Aminobenzylphosphonic Acid *(**2**)*

**Scheme 6:**
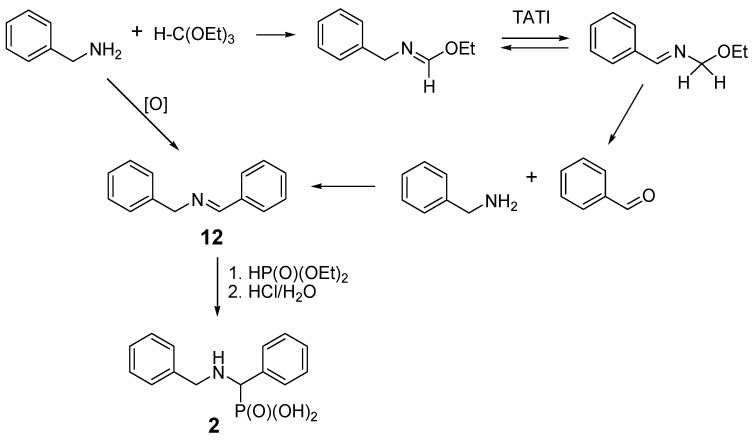
Possible route for the formation of *N*-benzylidenebenzylamine **12**.

Most likely, compound **2** is produced by the addition of diethyl phosphite to *N*-benzylidenebenzylamine **12**. The latter compound might be formed by a process already described in the literature, in which benzylamines undergo catalytic oxidative dehydrogenation yielding the appropriate Schiff bases [24,25]. Compound **12** is the presumed substrate for the production of compound **2** via the simple addition of phosphite [20]. Another possibility is the formation of benzaldehyde in the reaction of benzylamine with triethyl orthoformate followed by a process similar to the transamination–transimination (TATI) reaction [26]. The formed benzaldehyde then reacts with benzylamine yielding *N*-benzylidenebenzylamine **12** (Scheme 6). Taking into consideration all the observation and accounts given above, a mechanism of the whole process of aminophosphonate formation could be proposed (Scheme 6).

**Scheme 7:**
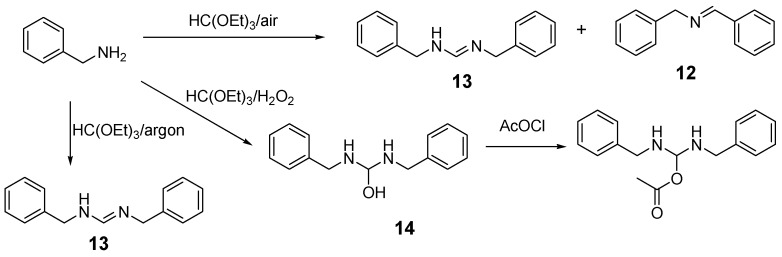
Reaction of benzylamine with triethyl orthoformate under anaerobic and aerobic conditions.

Thus, we decided to study the reaction of benzylamine with triethyl orthoformate (2:1 molar ratio) under anaerobic (under argon) and oxidative conditions (atmospheric conditions and conditions resulting from the addition of a small quantity of hydrogen peroxide to the reaction mixtures). Quite interestingly the reaction of benzylamine and triethyl orthoformate carried out under argon gave nearly exclusively *N*,*N*’-dibenzylimino formamide **13** as the product, the reaction under atmospheric conditions gave a complex mixture of compounds with compounds **12** and **13** as the major products, and the reaction carried out under the presence of hydrogen peroxide gave *N*,*N*’-dibenzylmethanol (compound **14**, Scheme 7), which was identified by spectroscopic methods and its conversion to the *O*-acetyl derivative. *N*,*N*’-Dibenzylmethanol did not react with diethyl phosphite at all.

**Scheme 8:**
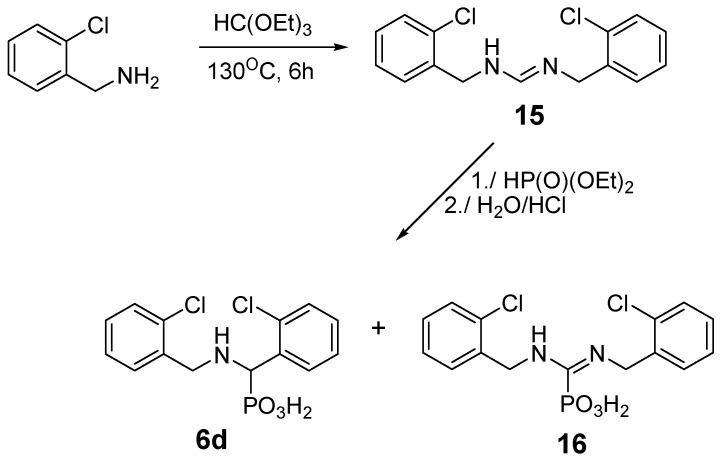
Reaction of *N,N’*-di(*o*-chlorobenzyl)imino formamide with diethyl phosphite.

The influence of the presence of oxygen on the course of the three-component reaction was also studied under standard conditions, namely, by heating the reagents at 135 °C for 12 h. Despite the applied conditions (under argon or by applying small quantities of hydrogen peroxide), reactions carried out using a stoichiometric ratio of the substrates (1:2:1) gave practically equimolar mixtures of products (aminomethylenebisphosphonate **1** and *N*-ethylated aminomethylenebisphosphonate (Similary to that shown on Scheme 5). The presence of significant quantities of hydrogen peroxide in the reaction medium resulted in the formation of *N*-benzyl aminobenzylphosphonic acid (**2**) in high (95%) yield. This supports the speculation that the oxidation of imines has a significant influence on the course of the reaction. It is also worth noting that the processes carried out under oxidative conditions additionally resulted in elevated ethylation of the formed phosphonates. Finally, reaction of *o*-chlorobenzylamine with triethyl orthoformate, provided the derivative of *N,N’*-di(*o*-chlorobenzyl)iminol formamide **15** obtained as the major product with satisfactory yield (31% yield). Reaction of this compound with diethyl phosphite (1:2 molar ratio) followed by the acid hydrolysis of the reaction mixture provided two products (30% total yield; Scheme 8), namely, amino phosphonate **6d** and a side-product that was not isolated from the reaction mixture. Based on the spectroscopic and MS data of the mixture of these products, the structure of **16** was tentatively proposed for this compound. Reaction carried out using a stoichiometric ratio of the substrates (1:2:1) gave aminomethylenephosphonic acid (compound **6d**) as the major product in the reaction of *o*-chlorobenzylamine (Table 1 entry 8).

## 3. Experimental Section

### 3.1. General Information

All solvents and reagents were purchased from commercial suppliers, were of analytical grade and were used without further purification. Unless otherwise specified, solvents were removed with a rotary evaporator. The ^1^H-, ^31^P- and ^13^C-NMR spectroscopic experiments were performed on a Bruker Avance II Ultrashield Plus (Bruker, Rheinstetten, Germany) operating at 600.58 MHz (^1^H), 243.12 MHz (^31^P{^1^H}) and 151.016 MHz (^13^C), or on Bruker Avance III 500 MH (Bruker, Rheinstetten, Germany) operating at 500.14 MHz (^1^H) and on Jeol JNM-ECZ 400S Research FT NMR Spectrometer (JEOL Ltd., Tokyo, Japan) operating at 399.78 MHz (^1^H), and 161.98 MHz (^31^P{^1^H}). Measurements were made in DMSO-*d*_6_, CDCl_3_ and solutions of D_2_O + NaOD at 300 K, and all solvents were supplied by ARMAR AG (Dottingen, Switzerland). Chemical shifts are reported in ppm relative to TMS and 85% H_3_PO_4_, used as external standards, and coupling constants are reported in Hz. Melting points were determined on an SRS Melting Point Apparatus OptiMelt MPA 100 (Stanford Research Systems, Sunnyvale, CA, USA) and are reported uncorrected. Mass spectra were recorded at the Faculty of Chemistry, Wroclaw University of Science and Technology using a Waters LCT Premier XE mass spectrometer (method of electrospray ionization, ESI) (Waters, Milford, MA, USA).

### 3.2. Crystallography

The single-crystal X-ray data were collected with a KM4 CCD four-circle diffractometer. The cell constant determination, data collection and data reduction were performed using the CrysAlis PRO software package [27]. The structure was solved and refined by the SHELXS-2013/1 and SHELXL-2014/7 programs, respectively [28,29]. All non-hydrogen atoms were refined with anisotropic displacement parameters. All hydrogen atoms were located in a difference Fourier map and refined freely. The structure was visualized with the Ortep-3 program [30]. The experimental and crystallographic data are included in the cif file deposited at the Cambridge Crystallographic Data Centre: CCDC 1409625. Crystal data. C_28_H_29_Cl_5_N_2_O_6_P_2_, M = 728.72, triclinic, *a* = 9.2663(4), *b* = 12.6722(6), *c* = 14.5066(7) Å, α = 98.233(4), β= 105.849(4), γ = 94.450(4)°, V = 1609.55(13) Å^3^, T = 299 K, space group = P${}^{\bar{1}}$, Z = 2, 20921 reflections measured, and 6340 unique reflections (*R_int_* = 0.0254) which were used in all calculations. The final *wR*^2^*(F*^2^*)* and *R*^1^*(F)* were 0.0970 (for all data) and 0.0389 (for 5454 observed data), respectively.

### 3.3. General Procedure for the Synthesis

A mixture of an amine (0.03 mol) and the appropriate amounts (see tables) of diethyl phosphite and triethyl orthoformate were either heated with stirring at an elevated temperature (80 °C or 135 °C) for 12 h on the heating plate of a Radley’s Carousel or placed in a microwave synthesizer (Biotage Initiator + SP Vave) for the time indicated in Table 1. Then, the volatile components of the reaction mixture were evaporated, and the resulting crude product was hydrolysed under reflux for 12 h with 6 N hydrochloric acid (20 mL). After evaporation, the crude products were obtained and purified by crystallization from water or water-ethanol mixtures if necessary.

*N-Benzylaminomethylenebisphosphonic acid* (**1**) was obtained as a white solid (68% yield); m.p. 231–234 °C (lit. m.p. = 246–249 °C [31]); ^31^P-NMR (243.12 MHz, D_2_O + NaOD, ppm): δ = 17.26; ^1^H-NMR (600.58 MHz, D_2_O, ppm): δ = 2.61 (1H, t, *J* = 17.56 Hz, C**H**P), 3.84 (2H, s, C**H**_2_), 7.19 (1H, t, *J* = 6.9 Hz, Ar), 7.27 (2H, t, *J* = 7.1 Hz, Ar), 7.32 (2H, d, *J* = 7.1 Hz, Ar); ^13^C-NMR (151.02 MHz, D_2_O + NaOD, ppm): δ = 54.21, 59.42 (t, *J* = 125.8 Hz, **C**P), 127.62, 128.74, 128.89, 138.51; HRMS (TOF MS ESI^−^): [M − H]^−^ Calcd for C_8_H_12_NO_6_P_2_: 280.0140, found: 280.0150.

*N-Benzylaminobenzylphosphonic acid* (**2**) was obtained as a white solid (53% yield); m.p. 227–228 °C (lit. m.p. = 236–237 °C [20]); ^31^P-NMR (243.12 MHz, D_2_O + NaOD, ppm): δ = 15.89; ^1^H-NMR (600.58 MHz, D_2_O, ppm): δ = 3.35 & 3.50 (2H, AB system, *J* = 11.2 Hz, CH_2_), 3.56 (1H, d, *J* = 19.4 Hz, C**H**P), 7.10–7.20 (4H, m, Ar), 7.21–7.26 (4H, m, Ar), 7.27–7.32 (2H, m, Ar); HRMS (TOF MS ESI^−^): [M − H]^−^ Calcd for C_14_H_16_NO_3_P: 276.0789, found: 276.0784.

*N-(2-Methylbenzylamino)methylenebisphosphonic acid* (**5a**) was obtained as a white solid (31% yield); m.p. 235–236°C decomp. (lit. m.p. = 256–257 °C [31]); ^31^P-NMR (243.12 MHz, D_2_O + NaOD, ppm): δ = 17.46; ^1^H-NMR (600.58 MHz, D_2_O + NaOD, ppm): δ = 2.03 (s, 3H, C**H**_3_), 2.43 (t, 1H, *J* = 16.9 Hz, C**H**P), 6.99 & 7.01 (AB system, 4H, *J* = 7.6 Hz, Ar); ^13^C-NMR (151.02 MHz, D_2_O + NaOD, ppm): δ = 18.20, 51.89, 59.31 (t, *J* = 128.6 Hz, **C**P), 125.86, 126.99, 128.19, 129.96, 136.50, 138.60; HRMS (TOF MS ESI^−^): [M − H]^−^ Calcd for C_9_H_15_NO_6_P_2_: 294.0296, found: 294.0302.

*N-(3-Methylbenzylamino)methylenebisphosphonic acid* (**5b**) was obtained as a white solid (36% yield); m.p. 248–250 °C; ^31^P-NMR (243.12 MHz, D_2_O + NaOD, ppm): δ = 17.10; ^1^H-NMR (600.58 MHz, D_2_O + NaOD, ppm): δ = 2.24 (s, 3H, C**H**_3_), 2.65 (t, 1H, *J* = 17.2 Hz, C**H**P), 3.85 (s, 2H, C**H**_2_), 7.06 (1H, d, *J* = 7.0 Hz), 7.14–7.25 (3H, m, Ar); ^13^C-NMR (151.02 MHz, D_2_O + NaOD, ppm): δ = 20.43, 54.48, 59.31 (t, *J* = 126.3 Hz, **C**P), 125.29, 127.49, 128.51, 128.99, 138.47, 140.59; HRMS (TOF MS ESI^−^): [M − H]^−^ Calcd for C_9_H_15_NO_6_P_2_: 294.0296, found: 294.0301.

*N-(4-Methylbenzylamino)methylenebisphosphonic acid* (**5c**) was obtained as a white solid (28% yield); m.p. 244–246 °C; ^31^P-NMR (243.12 MHz, D_2_O + NaOD, ppm): δ = 17.50; ^1^H-NMR (600.58 MHz, D_2_O + NaOD, ppm): δ = 2.04 (s, 3H, C**H**_3_), 2.45 (t, 1H, *J* = 16.8 Hz, C**H**P), 3.65 (s, 2H, C**H**_2_), 7.01 & 7.02 (AB system, 4H, *J* = 7.52 Hz, Ar); ^13^C-NMR (151.02 MHz, D_2_O + NaOD, ppm): δ = 20.15, 54.28, 59.39 (t, *J* = 194.1 Hz, **C**P), 128.50, 129.02, 136.93, 137.56; HRMS (TOF MS ESI^−^): [M − H]^−^ Calcd for C_9_H_15_NO_6_P_2_: 294.0296, found: 294.0301.

*N-(3-Methylbenzylamino)-3-methylbenzylphosphonic acid* (**6b**) was obtained as a white solid (19% yield); m.p. 150–157°C decomp.; ^31^P-NMR (243.12 MHz, D_2_O + NaOD, ppm): δ = 15.89; ^1^H-NMR (600.58 MHz, D_2_O + NaOD, ppm): δ = 2.19 (3H, s, C**H**_3_), 2.23 (3H, s, C**H**_3_), 3.40 & 3.42 (AB system, 2H, *J* = 13.5 Hz, C**H**_2_), 3.57 (d, 1H, *J* = 15.8 Hz, C**H**P), 6.94–6.99 (2H, m, Ar), 7.02 (2H, t, *J* = 9.25 Hz, Ar), 7.09–7.31 (4H, m, Ar); ^13^C-NMR (151.02 MHz, D_2_O + NaOD, ppm): δ = 20.39, 20.53, 51.22, 51.32, 62.96 (d, *J* = 134.2 Hz), 125.65, 126.05, 126.88, 127.91, 127.71, 128.51, 128.51, 129.42, 129.81, 137.76, 138.53, 139.44, 140.45; HRMS (TOF MS ESI^−^): [M − H]^−^ Calcd for C_16_H_20_NO_3_P: 304.1103, found: 304.1111.

*N-(4-Methylbenzylamino)-4-methylbenzylphosphonic acid* (**6c**) was obtained as a white solid (7% yield); m.p. 158–160 °C; ^31^P-NMR (243.12 MHz, D_2_O + NaOD, ppm): δ = 17.51; ^1^H-NMR (600.58 MHz, D_2_O + NaOD, ppm): δ = 0.78 (3H, s, C**H**_3_), 0.82 (3H, s, C**H**_3_), 2.53 & 2.72 (2H, AB system, *J* = 13.1 Hz, C**H**_2_), 2.93 (1H, d, *J* = 17.3 Hz, C**H**P), 5.68 & 5.69 (4H, system AB, *J* = 7.8 Hz, Ar), 5.79 (m, 4H, Ar); HRMS (TOF MS ESI^−^): [M − H]^−^ Calcd for C_16_H_20_NO_3_P: 304.1103, found: 304.1118. ^13^C-NMR was unclear because of the low solubility of **6c.**

*N-(2-Chlorobenzylamino)-2-chlorobenzylphosphonic acid* (**6d**) was obtained as a white solid (24% yield); m.p. 223–225 °C (lit. mp = 205–207 °C [32]); ^31^P-NMR (243.12 MHz, D_2_O + NaOD, ppm): δ = 15.26; ^1^H-NMR (600.58 MHz, D_2_O + NaOD, ppm): δ = 3.51 & 3.52 (2H, AB system, *J* = 30.0 Hz, CH_2_), 4.23 (1H, d, *J* = 19.3 Hz, CHP), 7.07–7.14 (4H, m, Ar), 7.21 (1H, t, *J* = 6.9 Hz, Ar), 7.22–7.27 (2H, m, Ar), 7.59 (1H, d, *J* = 8.1 Hz); ^13^C-NMR (151.02 MHz, D_2_O + NaOD, ppm): δ = 49.31, 39.41, 58.4 (d, *J* = 133.0 Hz, CP), 126.78, 127.16, 128.84, 129.05, 129.39, 129.91 129.94, 130.96, 133.57, 134.50, 136.65, 138.32; HRMS (TOF MS ESI^−^): [M − H]^−^ Calcd for C_14_H_14_Cl_2_NO_3_P: 344.0010, 345.9983 found: 344.0022, 346.0074.

*N-(3-Chlorobenzylamino)-3-chlorobenzylphosphonic acid* (**6e**) was obtained as a white solid (31% yield); m.p. 196–199 °C decomp.; ^31^P-NMR (243.12 MHz, D_2_O + NaOD, ppm): δ = 15.11; ^1^H-NMR (600.58 MHz, D_2_O, ppm): δ = 3.31 & 3.34 (2H, AB system, *J* = 13.6 Hz, C**H**_2_), 3.44 (1H, d, *J* = 17.9 Hz, C**H**P), 6.91 (s, 1H, Ar), 6.95 (s, 1H, Ar), 7.04–7.07 (m, 5H, Ar), 7.16 (s, 1H, Ar); ^13^C-NMR (151.02 MHz, D_2_O + NaOD, ppm): δ = 51.26, 51.35, 62.99 (d, *J* = 133.0 Hz, **C**P), 126.20, 127.04, 127.35, 128.59, 128.61, 128.64, 129.31, 129.91, 133.00, 133.44, 141.42, 142.91; HRMS (TOF MS ESI^−^): [M − H]^−^ Calcd for C_14_H_14_Cl_2_NO_3_P: 344.0010, 345.9983 found: 344.0016, 345.9978.

*N-(4-Chlorobenzylamino)-4-chlorobenzylphosphonic acid* (**6f**) was obtained as a white solid (24% yield); m.p. 262–263 °C. ^31^P-NMR (243.12 MHz, DMSO-*d*_6_, ppm): δ = 10.32; ^1^H-NMR (600.58 MHz, DMSO-*d*_6,_ ppm): δ = 6.03 & 6.07 (2H, system AB, *J* = 13.93 Hz), 4.13 (1H, d, *J* = 14.391 Hz), 7.39–7.49 (8H, m); HRMS (TOF MS ESI^−^): [M − H]^−^ C_14_H_14_Cl_2_NO_3_P required 344.0010, 345.9983 found 344.0005, 345.9965. ^13^C-NMR was unclear because of the low solubility of **6f**.

*N-(3-Bromobenzylamino)methylenephosphonic acid* (**5h**) was obtained as a white solid (88% yield); m.p. 230–232 °C decomp. ^31^P-NMR (243.12 MHz, D_2_O + NaOD, ppm): δ = 18.55; ^1^H-NMR (600.58 MHz, D_2_O + NaOD, ppm): δ = 2.12 (t, 1H, *J* = 16.82 Hz, C**H**P), 3.39 (s, 2H, C**H**_2_), 6.79 (1H, t, *J* = 7.7 Hz, Ar), 6.88 (1H, d, *J* = 7.7 Hz, Ar), 6.94 (1H, d, *J* = 7.7 Hz, Ar), 7.08 (1H, s, Ar); ^13^C-NMR (100.61 MHz, D_2_O + NaOD, ppm): δ = 53.65, 58.74 (t, *J* = 118.9 Hz, **C**P_2_), 121.00, 127.05, 129.58, 130.33, 130.94, 142.91; HRMS (TOF MS ESI^−^): [M − H]^−^ Calcd for C_8_H_12_BrNO_6_P_2_: 359.9226, found: 359.9227.

*N-(2-Thiophenylamino)-thiophen-2-ylmethylphosphonic acid* (**4**) was obtained as a white solid (54% yield); m.p. 215–216 °C decomp. ^31^P-NMR (243.12 MHz, D_2_O + NaOD, ppm): δ = 14.41; ^1^H-NMR (600.58 MHz, D_2_O + NaOD, ppm): δ = 3.78 & 3.85 (AB system, 2H *J* = 14.02 Hz, C**H**_2_), 6.00 (1H, d, *J* = 11.53 Hz, C**H**P ), 6.90–6.96 (4H, m, Ar), 7.27 (2H, bs, Ar); ^13^C-NMR (100.53 MHz, D_2_O + NaOD, ppm): δ = 45.45, 57.93 (d, *J* = 137.4 Hz, **C**HP), 124.45, 125.35, 126.45, 126.52, 126.53, 126.63, 127.21, 142.28; HRMS (TOF MS ES^−^): [M − H^+^], Calcd for C_10_H_12_NO_3_PS_2_ 287.9918, found 287.9912.

*Aminomethylenebisphosphonic acid* (**3**) was obtained as a white solid (49% yield in reaction with α-methylbenzylamine, or 85% yield in reaction with *p*-aminobenzylamine); mp 258-260°C decomp. (lit. mp = 250–265 °C decomp. [31,33]); ^31^P-NMR (243.12 MHz, D_2_O + NaOD, ppm): δ = 18.12; ^1^H-NMR (600.58 MHz, D_2_O + NaOD, ppm): δ = 2.63 (1H, t, *J* = 17.9 Hz, C**H**P), ^13^C-NMR (151.02 MHz, D_2_O + NaOD, ppm): δ = 50.80 (t, *J* = 127.9 Hz, **C**P); HRMS (TOF MS ESI^−^): [M − H]^−^ Calcd for CH_7_NO_6_P_2_: 189.9670, found:189.9660.

*N-(1-Phenylmeth-1-ylamino)methylenebisphosphonic acid* (**8**) was obtained as a white solid; (13% yield); m.p. 264−266 °C decomp. (lit. mp. = 251–253 °C [31]); ^31^P-NMR (161.98 MHz, D_2_O + NaOD, ppm): δ = 9.37; ^1^H-NMR (399.78 MHz, D_2_O + NaOD, ppm): δ = 1.12 (d, 3H, *J* = 6.60 Hz, C**H**_3_), 2.55 (t, 1H, *J* = 16.76 Hz, C**H**P_2_), 4.47–4.53 (q, 1H, *J*_1_ = 6.47 Hz, *J*_2_ = 18.27 Hz, C**H**NH), 7.15–7.21 (m, 1H, Ar), 7.27–7.38 (m, 4H, Ar); ^13^C-NMR (151.02 MHz, D_2_O + NaOD, ppm):δ = 21.40, 55.62 (dd, *J*_1_ = 123.32 Hz, *J*_2_ = 123.26 Hz, **C**P_2_), 56.64 (bs) ,127.30, 127.59, 128.77, 143.00; HRMS (TOF MS ES+): [M + H]^+^ Calcd for C_9_H_15_NO_6_P_2_: 296.0453, found: 296.0464.

*N-(3-Amino)benzylaminomethylenebisphosphonic acid* (**9b**) was obtained as a green solid; (24% yield); m.p. 240–242 °C; ^31^P-NMR (243.12 MHz, D_2_O + NaOD, ppm): δ = 17.18; ^1^H-NMR (500.14 MHz, D_2_O + NaOD, ppm): δ = 2.56 (t, 1H, *J* = 19.08 Hz, C**H**P_2_), 3.72 (s, 2H, CH_2_), 6.45 (bs, Ar), 6.67 (1H, d, *J* = 6.90 Hz, Ar), 6.72 (1H, d, *J* = 6.40 Hz, Ar), 7.04 (1H, t, *J* = 8.15 Hz, Ar); HRMS (TOF MS ESI^−^): [M − H]^−^ Calcd for C_8_H_14_N_2_O_6_P_2_: 295.0249, found: 294.9994.

*N*,*N*’*-Dibenzylmethanol* (**14**) was obtained as a white solid; (62% yield); m.p. 125 °C; ^1^H-NMR (600.58 MHz, CDCl_3_, ppm): δ = 4.38 (d, 4H, *J* = 14.71 Hz, **C**H_2_), 4.82 (s, 1H, **C**HNH), 7.25–7.29 (m, 6H, Ar), 7.31–7.34 (m, 2H), 8.12 (s, 1H, O**H**); HRMS (TOF MS ESI^−^): [M + H]^+^ Calcd for C_15_H_18_N_2_O_2_: 243.1497, found: 243.1175.

*O-Acethyl-N*,*N*’*-dibenzylmethanol* was obtained as a orange oil; (89% yield); ^1^H-NMR (600.58 MHz, CDCl_3_, ppm): δ = 2.37 (s, 4H, **C**H_2_), 4.88 (s, 1H, **C**H_3_), 7.23–7.27 (m, 4H, Ar), 7.29–7.35 (m, 6H, Ar) 9.26 (s, 1H, C**H**NH); HRMS (TOF MS ESI^−^): [M + H]^+^ Calcd for C_17_H_20_N_2_O_2_: 285.1603, found: 285.1612.

All the spectral data see supplementary information.

## 4. Conclusions

The analysis of the general mechanism of three component condensation of aminomethylenebisphosphonate formation was carried out using structurally variable benzylamines as the nucleophilic substrate. The amines was proven to be a valuable tool to follow appearance of various intermediates, non-phosphorus and phosphorus containing. They were isolated, their structures were identified and characterized which in turn allowed the suggestion the overall reaction route. The synthetic pathway leads to aminomethylenebisphosphonates via a series of imine and phosphonate products. The aforementioned factor can effect, however, with some difficulties in the final product separation. Since the three-component reaction usually yields a complex mixture of products that are difficult to separate, the resulting esters are not isolated. Instead, the crude reaction mixture is hydrolysed, yielding the bisphosphonic acid, which is subsequently isolated.

The three-component reaction between amines, triethyl orthoformate and diethyl phosphite is the most commonly used route for the preparation of *N*-substituted aminomethylenebisphosphonic acids. Surprisingly, benzylamines, depending on the reaction conditions, afforded the desired bisphosphonates or aminophosphonates or mixtures of the two. The studies presented above indicate that this is a complex and unpredictable reaction, which is strongly dependent on the applied conditions (ratio of reagents, presence of oxygen and temperature). However, manipulating the molar ratio of the reagents and the presence of oxygen can be used to control the course of the reaction.

Addition of greater excess of diethyl phosphite and increasing the reaction time caused visible enhancement of the final product yield while **2** disappeared immediately. However, the obvious conclusion that the increase in the yield of target bisphosphonate should be achieved by increase of molar ratio of diethyl phosphate used is not so apparent when considering that this usually causes substantial difficulties in separation of pure final products. Formation of compound **6d** was unambiguously shown by X-ray studies.

## Figures and Tables

**Table 1:** Products of the reactions between benzylamine, triethyl orthoformate and diethyl phosphite.

Entry	Amine: Phosphite: Orthoformate Molar Ratio	Reaction Conditions	Molar Amounts of Products (1:2) *	Total Isolated Yield (%)
1	1:2:1	80 °C, 12 h	0:100	53
2	1:2:1	135 °C, 12 h	43:57	10.5
3	1:2:1	reflux with removal of ethanol/12 h	65:35	20
4	1:2:1	80 °C, 6 min, MW		NR
5	1:2:1	80 °C, 12 min, MW	0:100	11.5
6	1:2:1	135 °C, 6 min, MW	0:100	5
7	1:2:1	135 °C, 12 min, MW	0:100	13.5
8	1:4:2	135 °C, 12 h	100:0	68

* determined basing on ^31^P-NMR spectra of separated products.

**Table 2:**
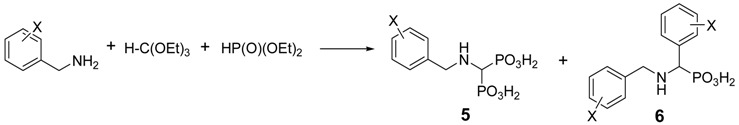
Products of the reactions between substituted benzylamines, triethyl orthoformate and diethylphosphite.

Entry	Substituent (X)	Product(s)	Amine:Phosphite:Orthoformate Molar Ratio	Molar Amounts of Products (5:6) *	Total Isolated Yield (%)
1	*o*-CH_3_	**6a**	1:2:1	**	
2	*o*-CH_3_	**5a**	1:4:2	100:0	31
3	*m*-CH_3_	**6b**	1:2:1	0:100	19
4	*m*-CH_3_	**5b**	1:4:2	100:0	36
5	*p*-CH_3_	**6c**	1:2:1	85:15	7
6	*p*-CH_3_	**5c**	1:4:2	100:0	28
7	*p*-CH_3_	**5c + 6c**	1:2:1 (80 °C)	0:100	13
8	*o*-Cl	**6d**	1:2:1	0:100	24
9	*m*-Cl	**6e**	1:2:1	0:100	31
10	*p*-Cl	**6f**	1:2:1	0:100	24
11	*p*-Cl	**5f + 6f**	1:4:2	57:43	75
12	*p*-F	**5g + 6g**	1:2:1	26:74	53
13	*m*-Br	**5h + 6h**	1:2:1	36:64	33
14	*m*-Br	**5h**	1:4:2	100:0	88
15	*o*-NO_2_	**6i**	1:2:1		NR

* determined basing on ^31^P-NMR spectra of separated products. ** Additional product **7** was observed (see text). NR—no reaction products detected.

## Supplementary Materials

^1^H-NMR, ^13^C-NMR, ^31^P-NMR, IR and MS spectra of representative compounds are available on line.
