# Supplementary material for: Three-Component Reaction of Benzylamines, Diethyl Phosphite and Triethyl Orthoformate: Dependence of the Reaction Course on the Structural Features of the Substrates and Reaction Conditions
Source: Molecules. 2017 Mar 11;22(3):450. doi: 10.3390/molecules22030450 (PMC6155184; doi:10.3390/molecules22030450)
Supplement: Supplementary file 1 [file molecules-22-00450-s001.pdf]

Supporting information

Three-component reaction of benzylamines, diethyl phosphite and triethyl orthoformate: Dependence of the reaction course on the structural features of the substrates and reaction conditions

Patrycja Miszczyk[1], I. Turowska-Tyrk[2], Paweł Kafarski[1], Ewa Chmielewska[1,*]

[a] Department of Bioorganic Chemisty, Faculty of Chemistry, Wrocław University of Science and Technology, Wybrzeże Wyspiańskiego 27, 50-370 Wrocław

[b] The Advanced Materials Engineering and Modelling Group, Faculty of Chemistry, Wrocław University of Science and Technology, Wybrzeże Wyspiańskiego 27, 50-370 Wrocław

^{31}P NMR, ^{1}H NMR, ^{13}C NMR and MS spectra for representative compounds and the mixture of reactions

compound **1**

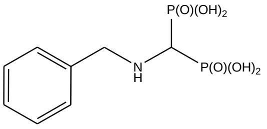

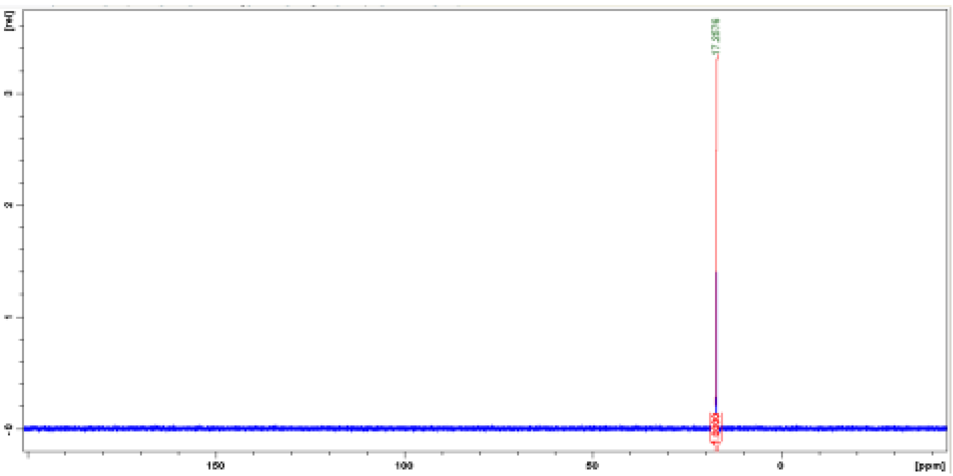

Figure S1. ^{31}P NMR spectrum of compound **1**

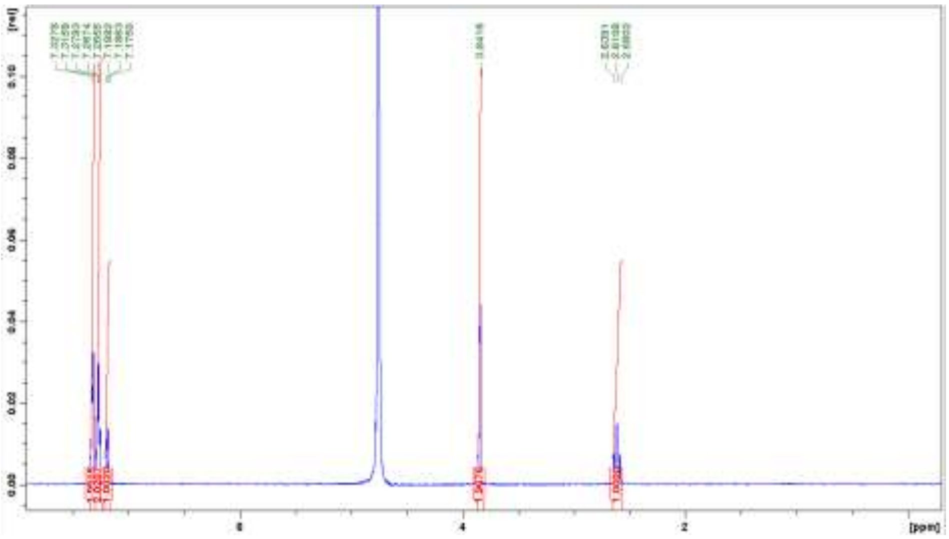

Figure S2. ^{1}H NMR spectrum of compound **1**

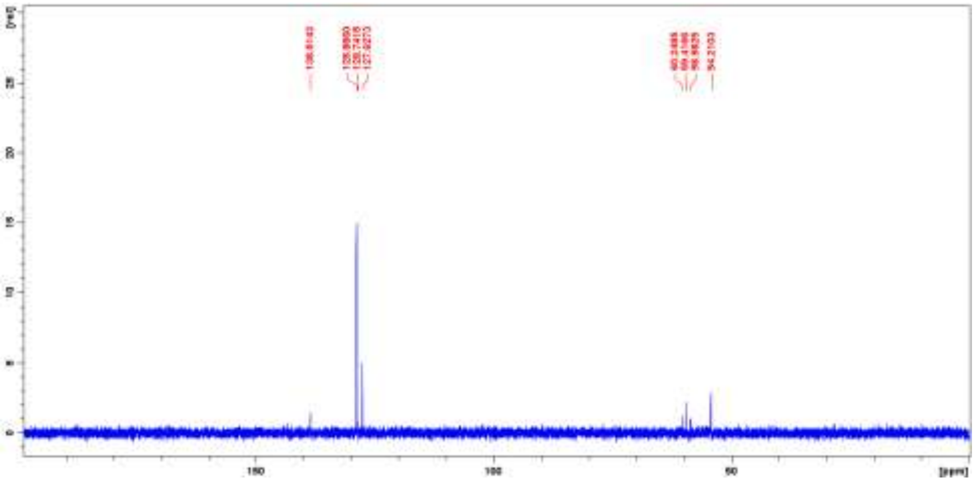

Figure S3. ^{13}C NMR spectrum of compound **1**

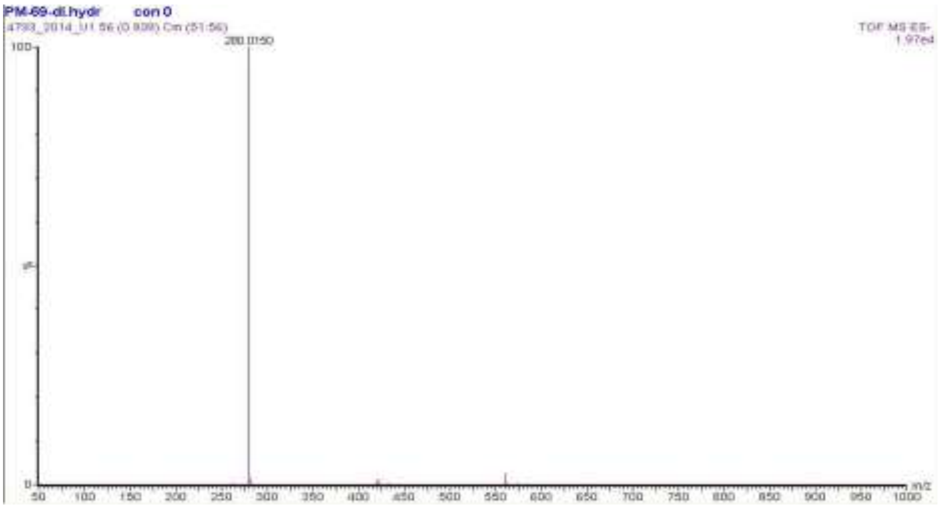

Figure S4. HRMS spectrum of compound **1**

compound **2** 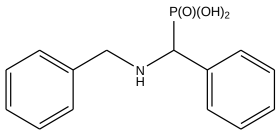

^{31}P NMR

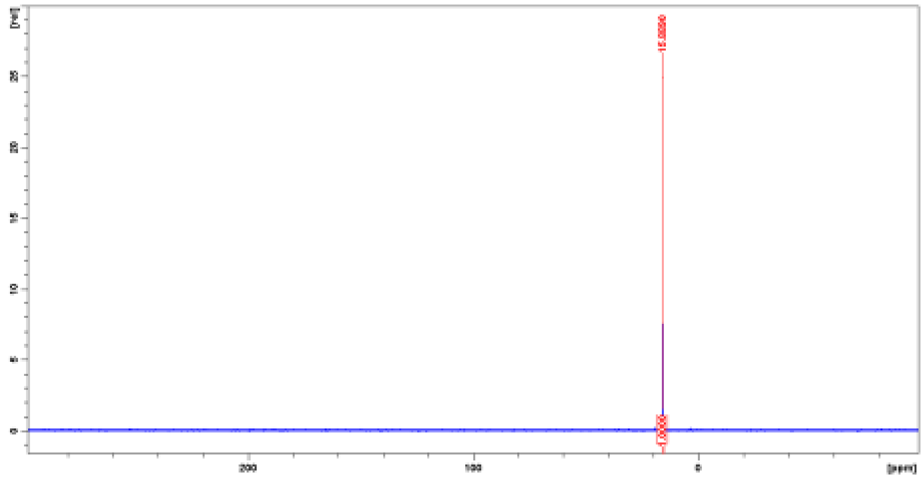

Figure S5. ^{31}P NMR spectrum of compound **2**

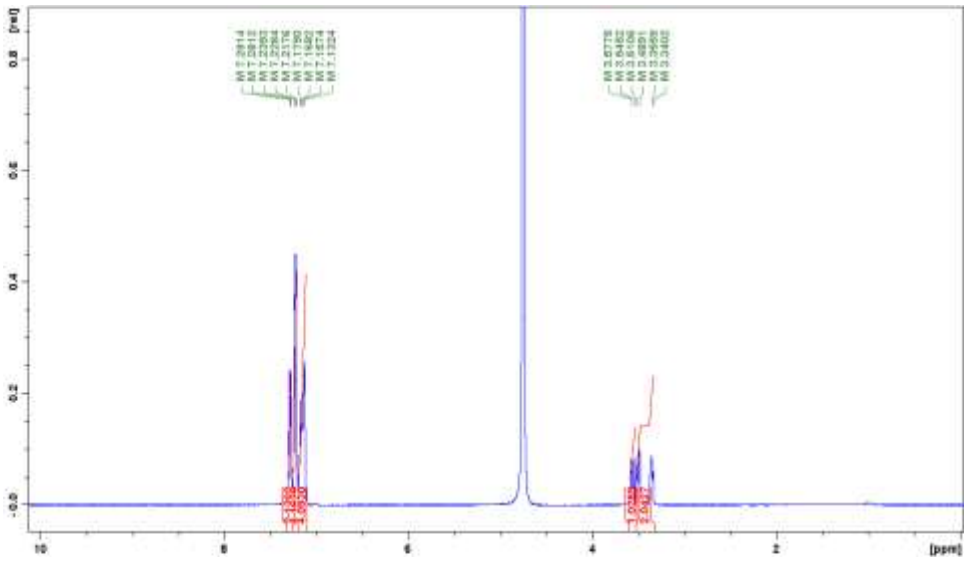

Figure S6. ^{1}H NMR spectrum of compound **2**

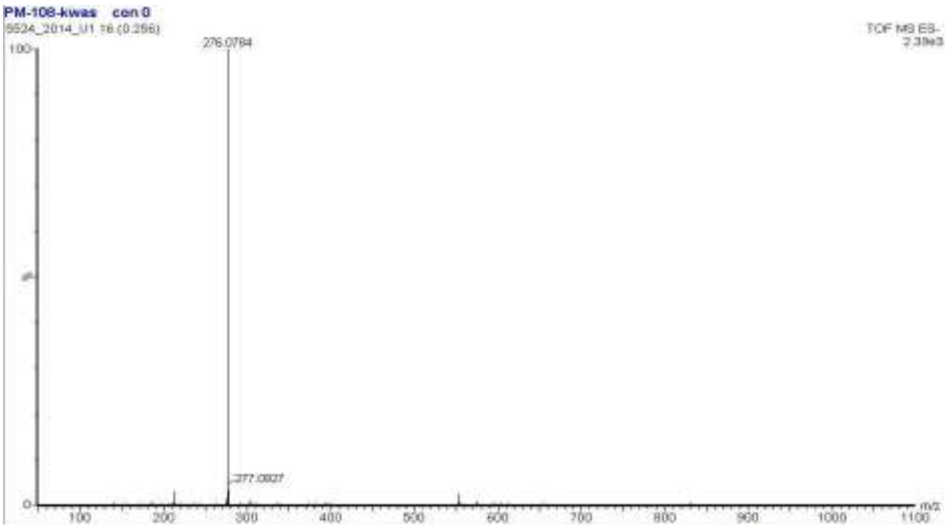

Figure S7. HRMS spectrum of compound **2**

mixture of compounds **1** and **2**

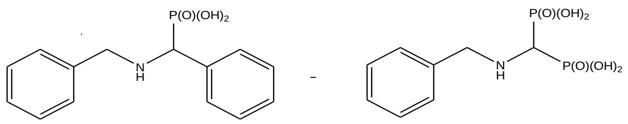

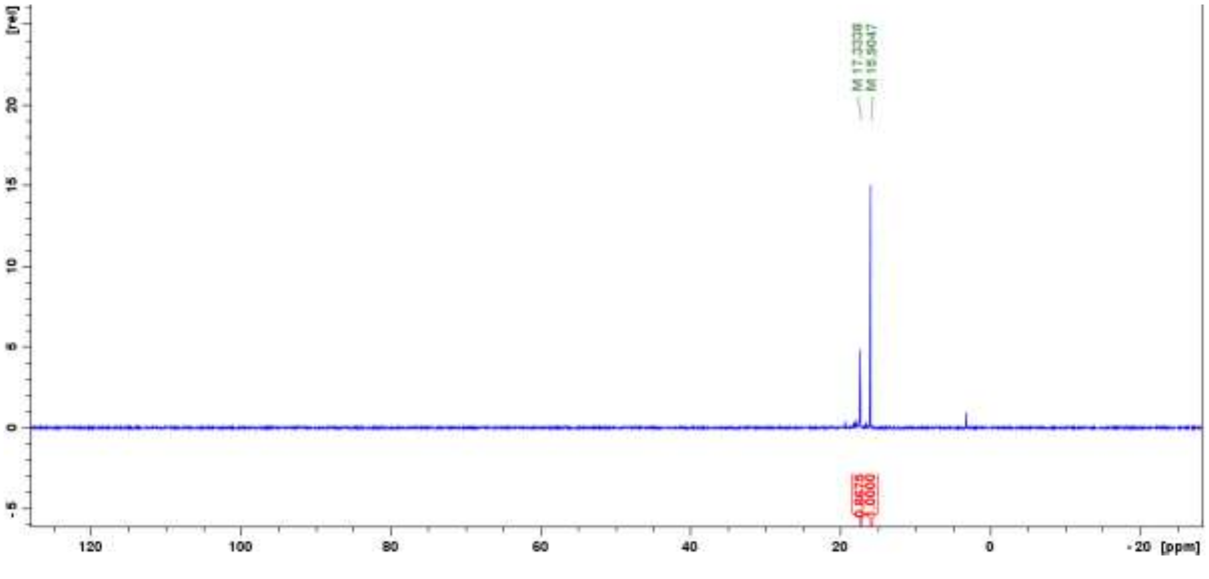

Figure S8. ^{31}P NMR spectrum of mixture of compounds **1** and **2**

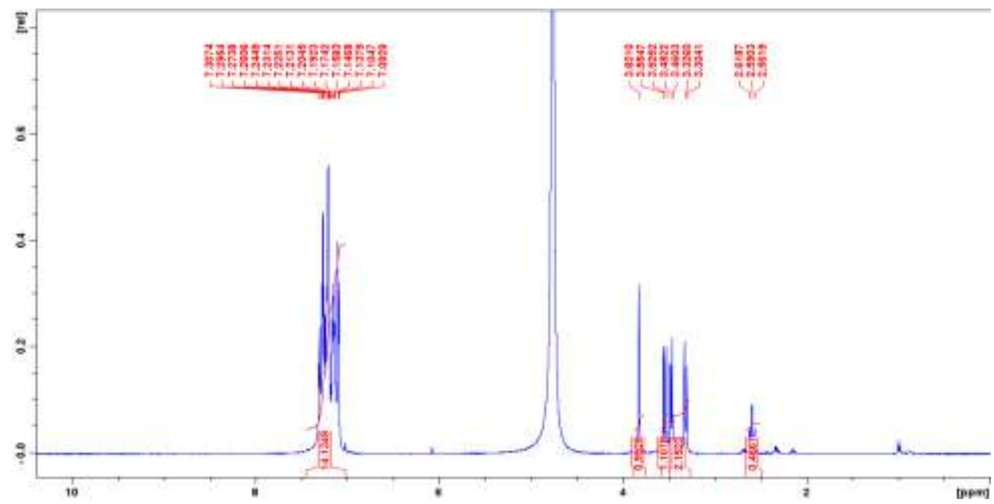

Figure S9. ^{1}H NMR spectrum of mixture of compounds **1** and **2**

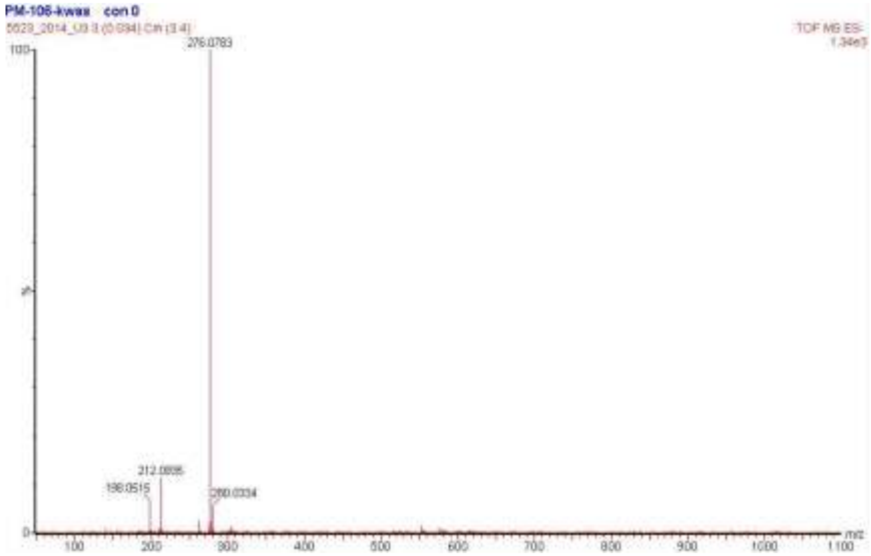

Figure S10. HRMS spectrum of mixture of compounds **1** and **2**

compound **3** 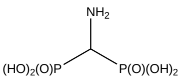

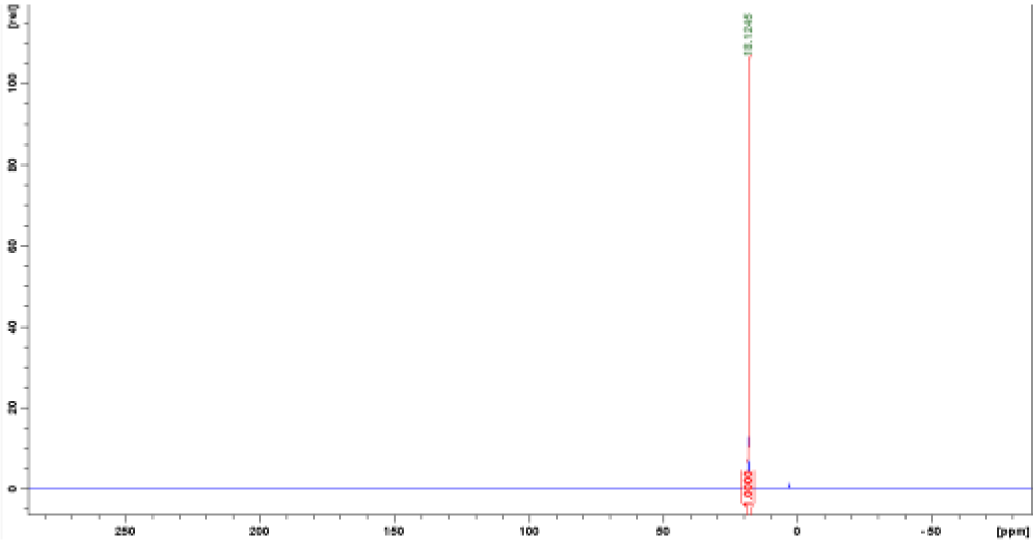

Figure S11. ^{31}P NMR spectrum of compound **3**

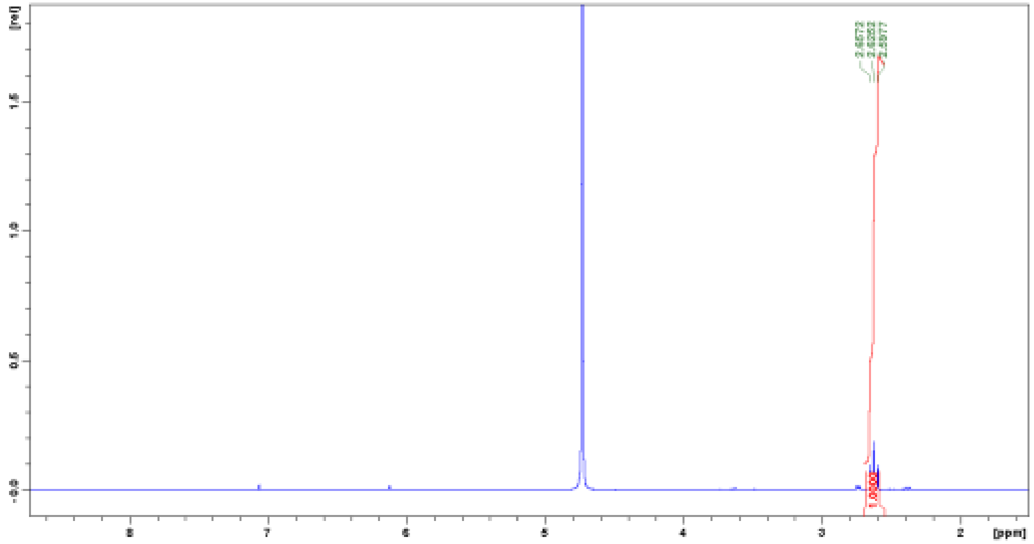

Figure S12. ^{1}H NMR spectrum of compound **3**

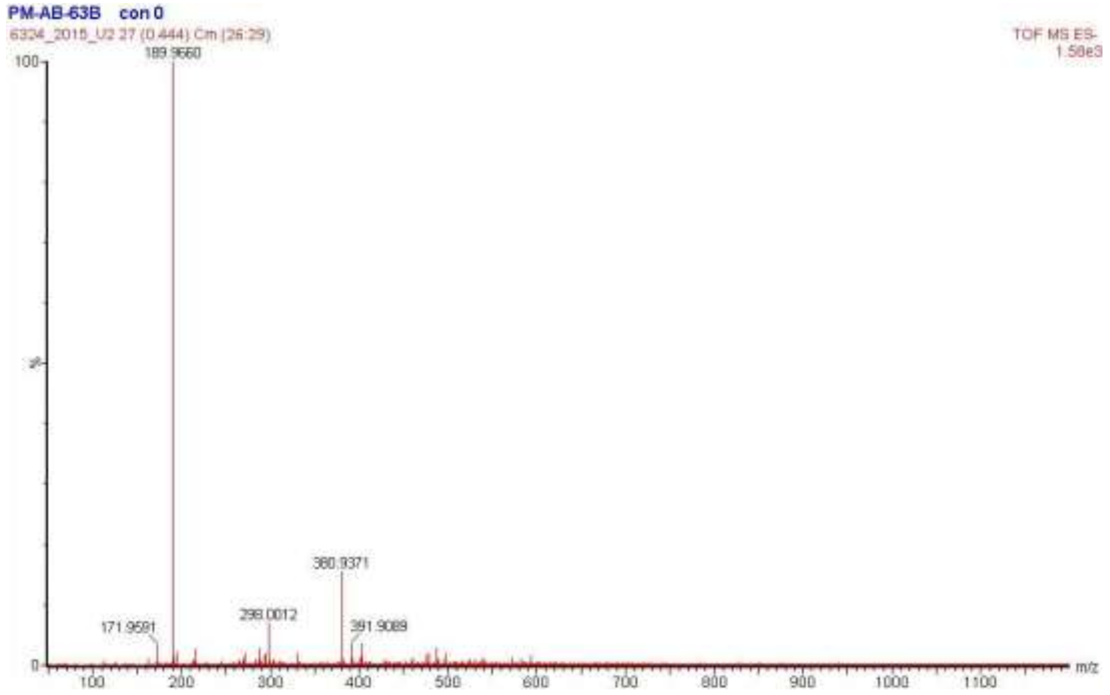

Figure S13. HRMS spectrum of compound **3**

compound **4**

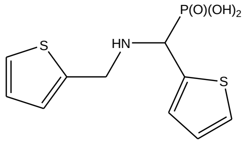

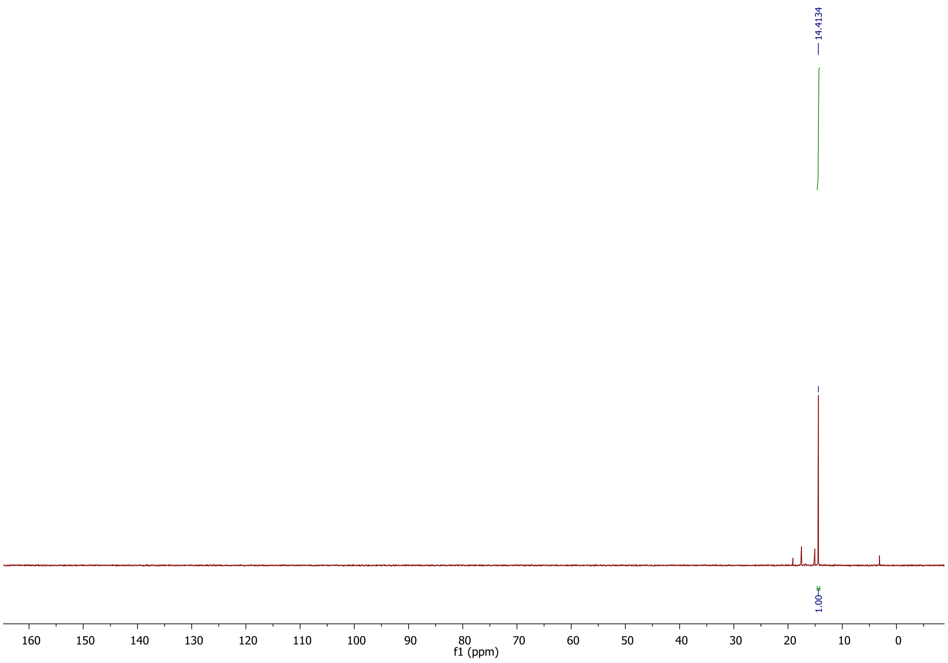

Figure S14. ^{31}P NMR spectrum of compound **4**

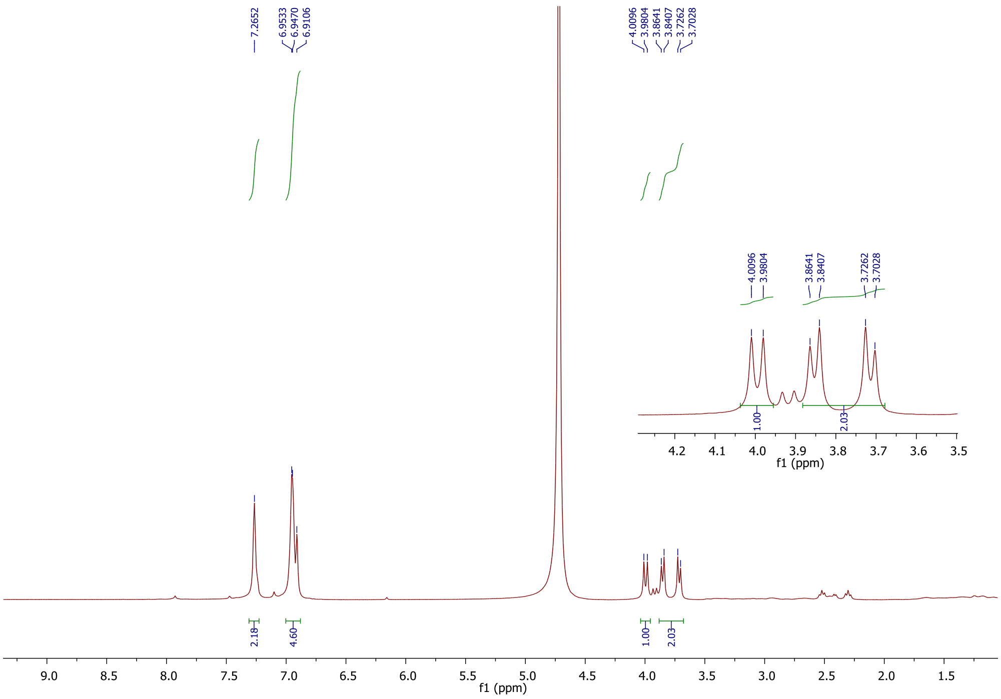

Figure S15. ^{31}H NMR spectrum of compound **4**

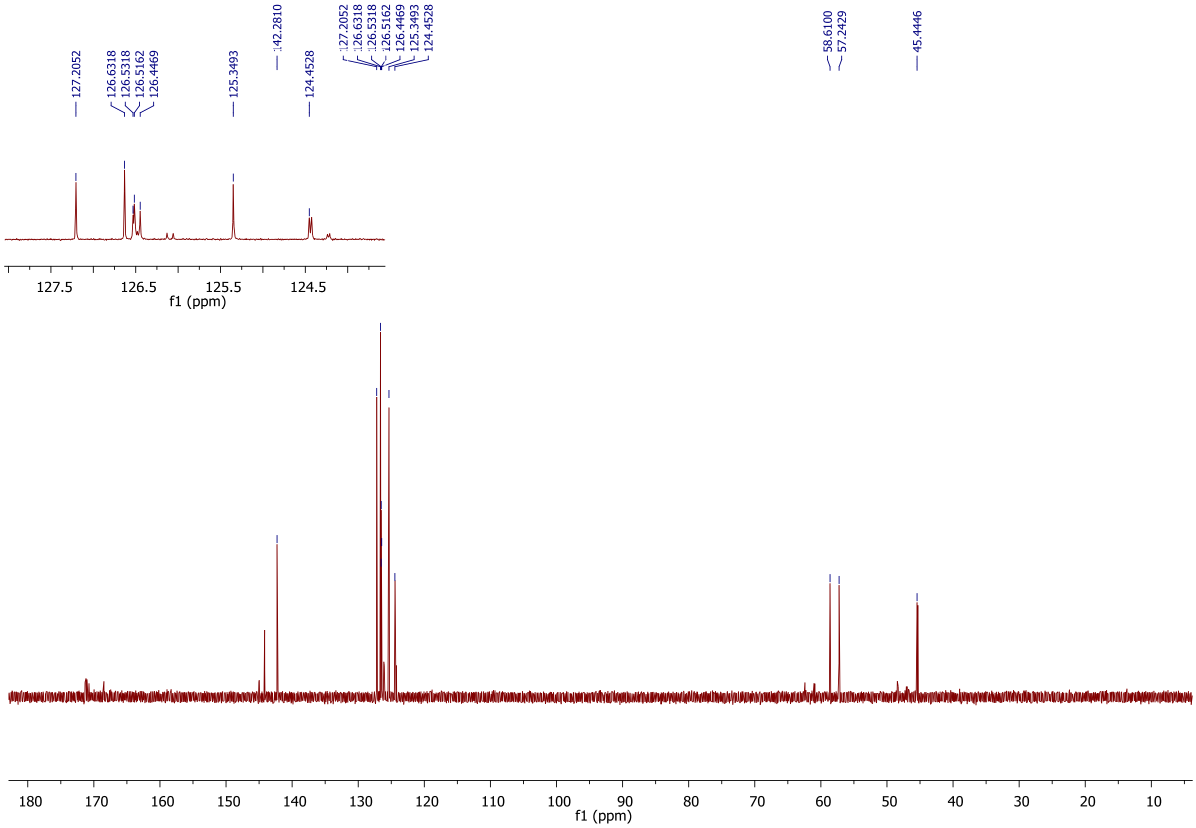

Figure S16. ^{13}C NMR spectrum of compound **4**

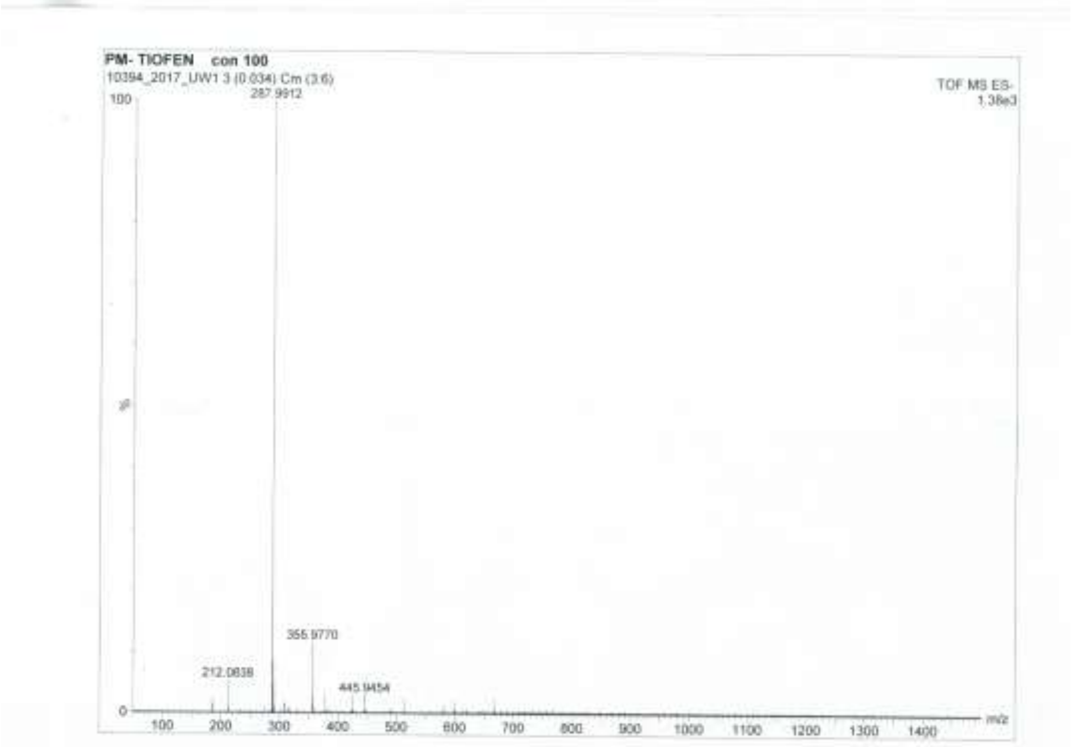

Figure S17. HRMS spectrum of compound **4**

compound **5a**

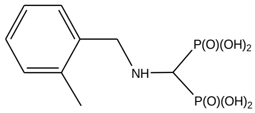

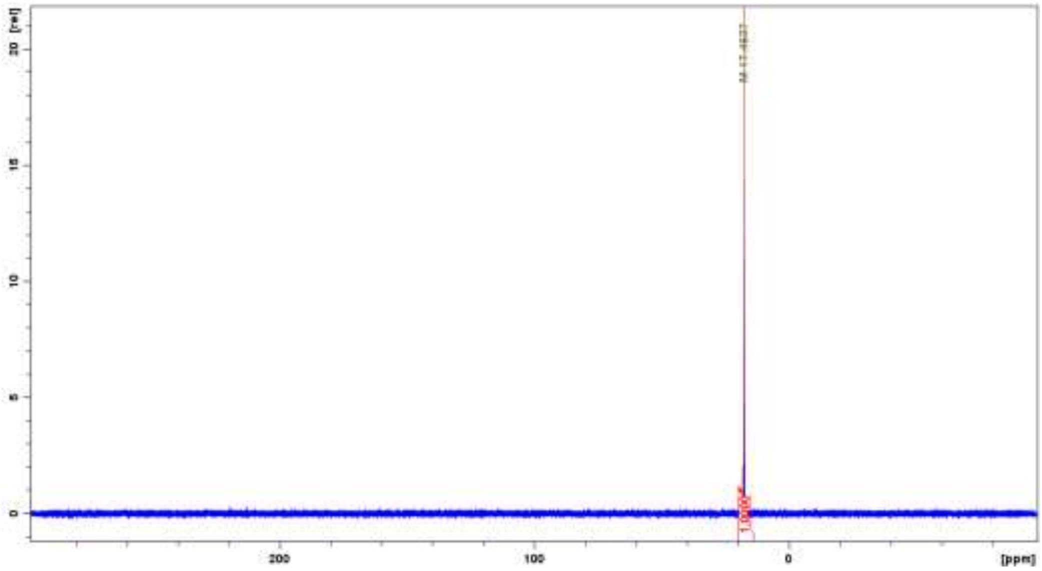

Figure S18. ^{31}P NMR spectrum of compound **5a**

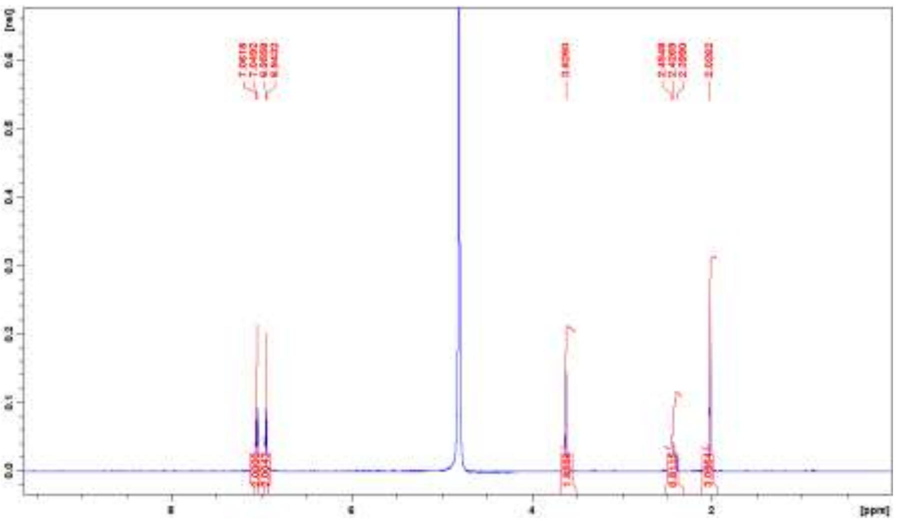

Figure S19. ^{1}H NMR spectrum of compound **5a**

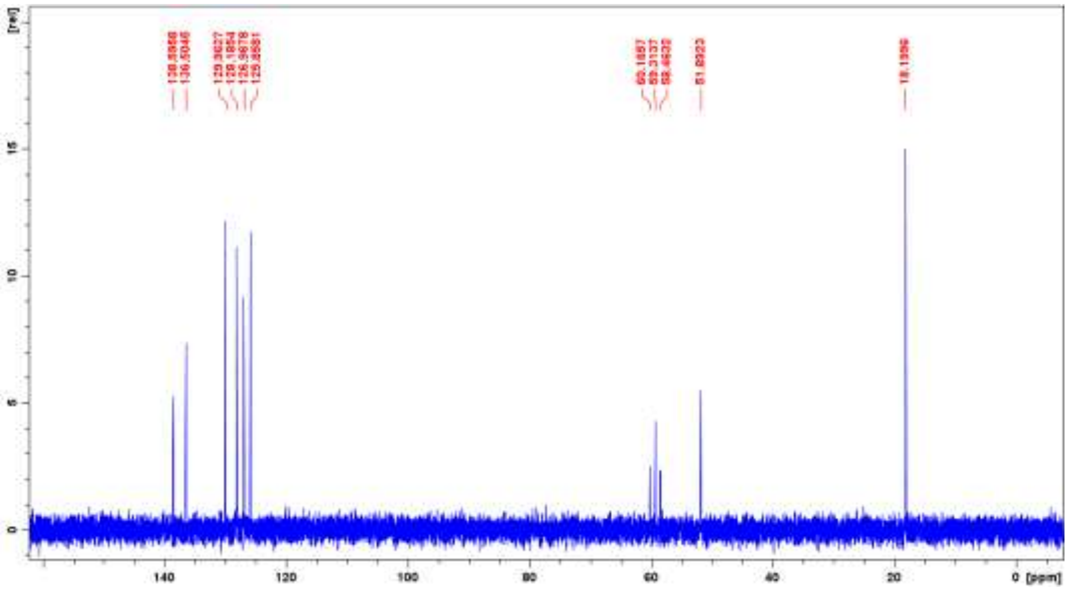

Figure S20. ^{13}C NMR spectrum of compound **5a**

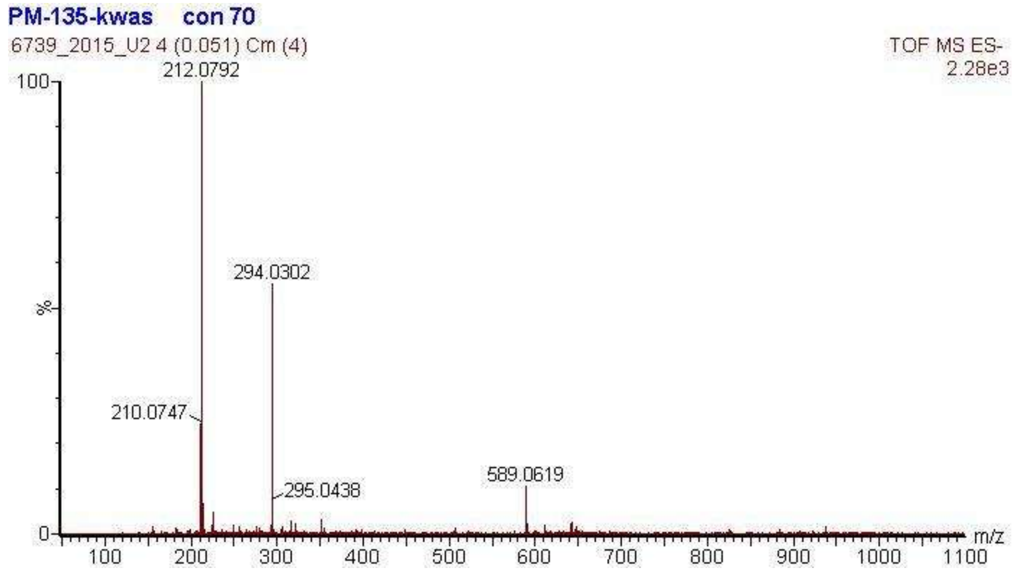

Figure S21. HRMS spectrum of compound **5a**

mixture of compounds **5a** and **6a**

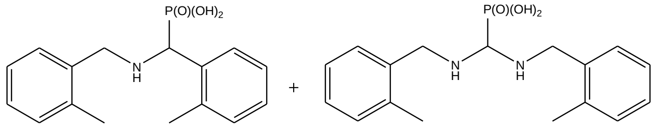

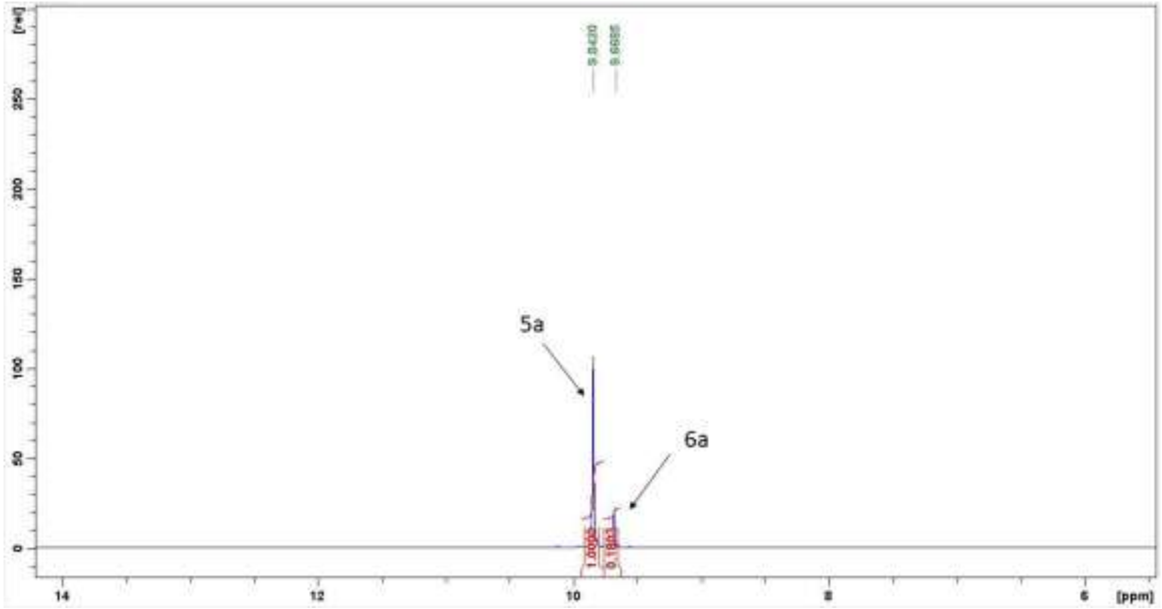

Figure S22. ^{31}P NMR spectrum of mixture of compounds **5a** and **6a**

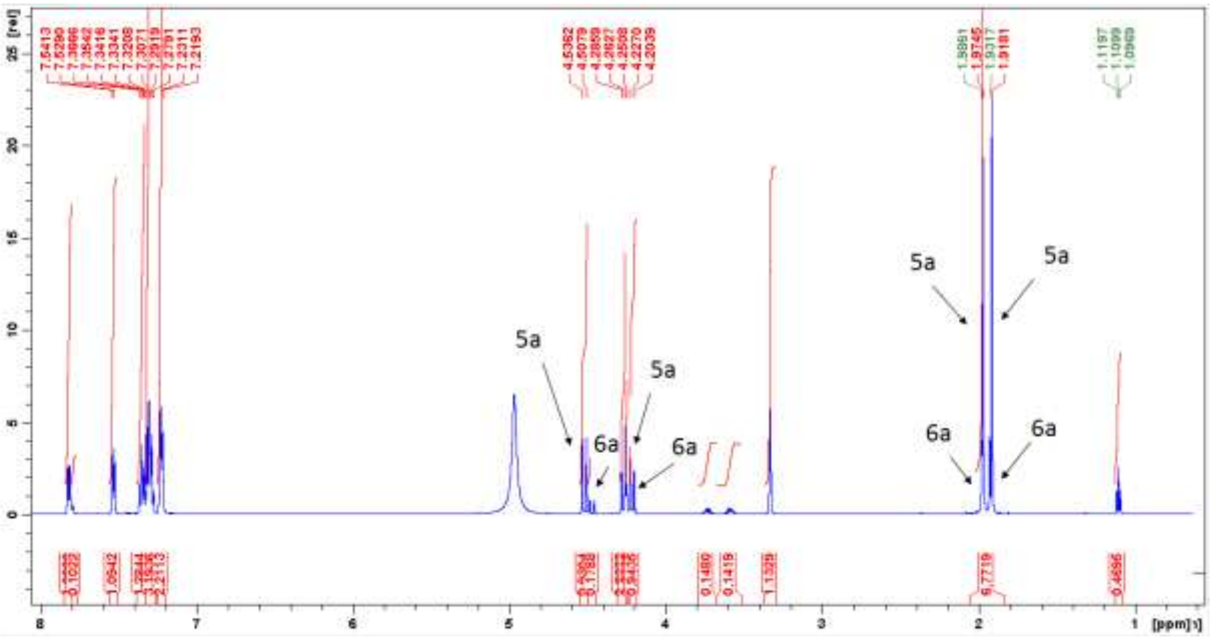

Figure S23. ^{1}H NMR spectrum of mixture of compounds **5a** and **6a**

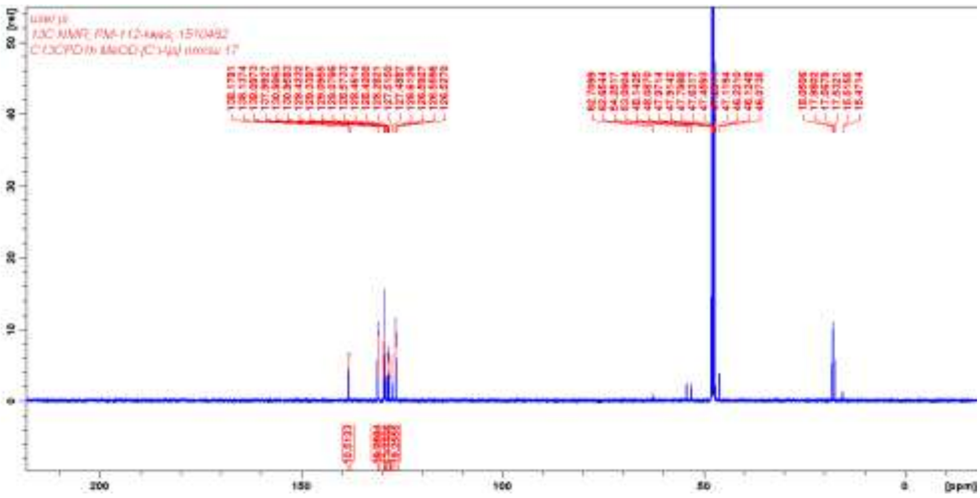

Figure S24. ^{13}C NMR spectrum of mixture of compounds **5a** and **6a**

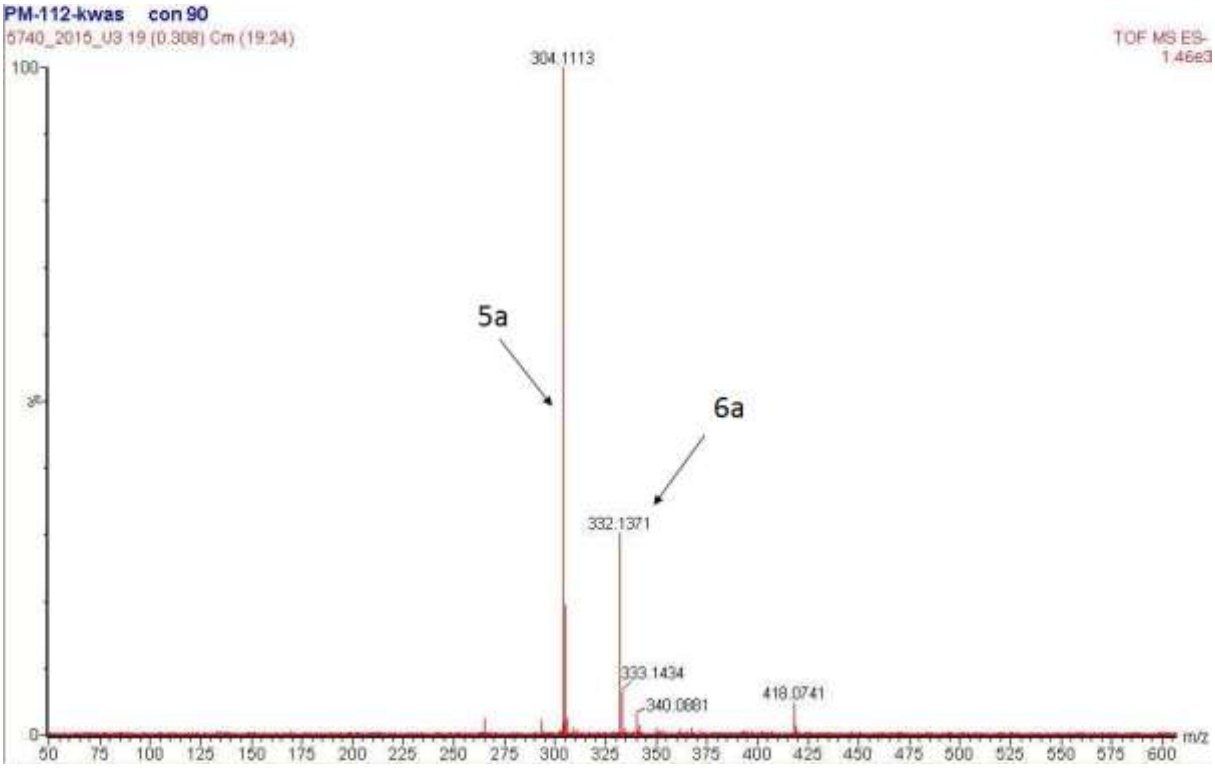

Figure S25. HRMS spectrum of mixture of compounds **5a** and **6a**

compound **5b**

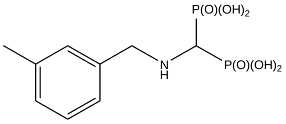

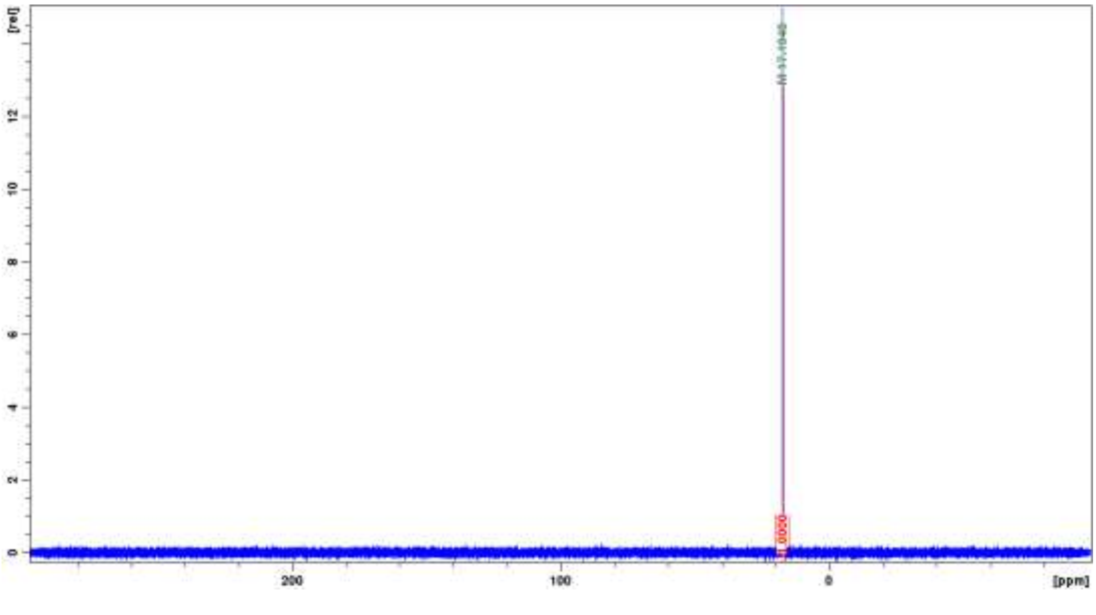

Figure S26. ^{31}P NMR spectrum of compounds **5b**

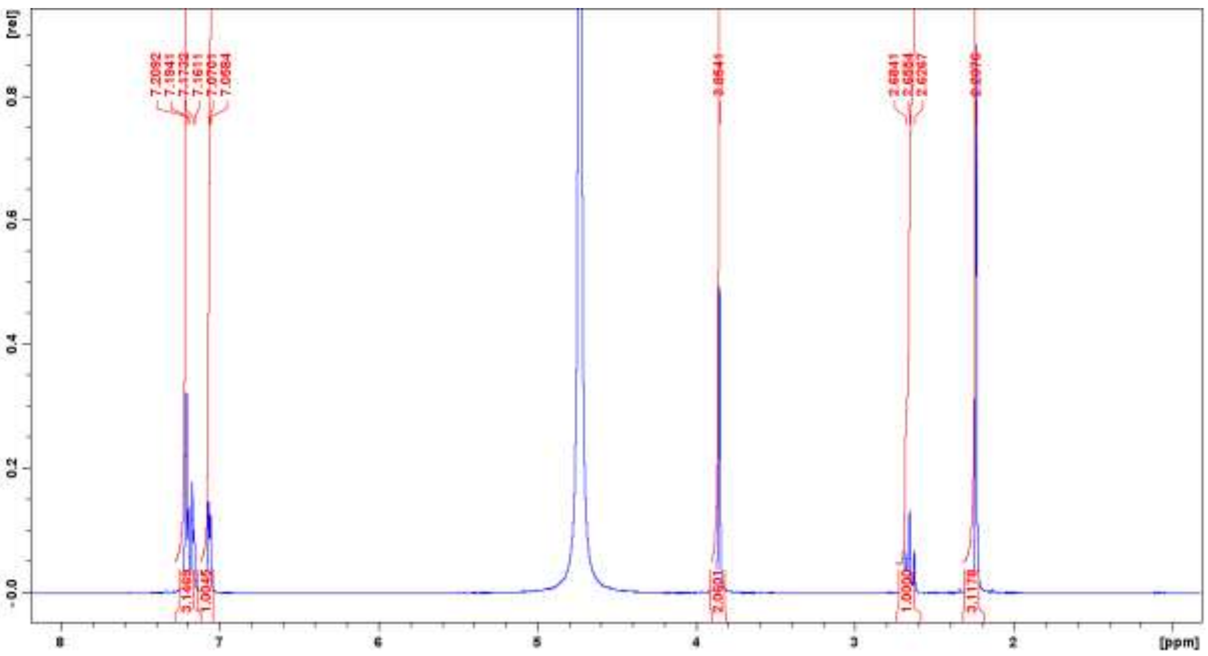

Figure S27. ^{1}H NMR spectrum of compounds **5b**

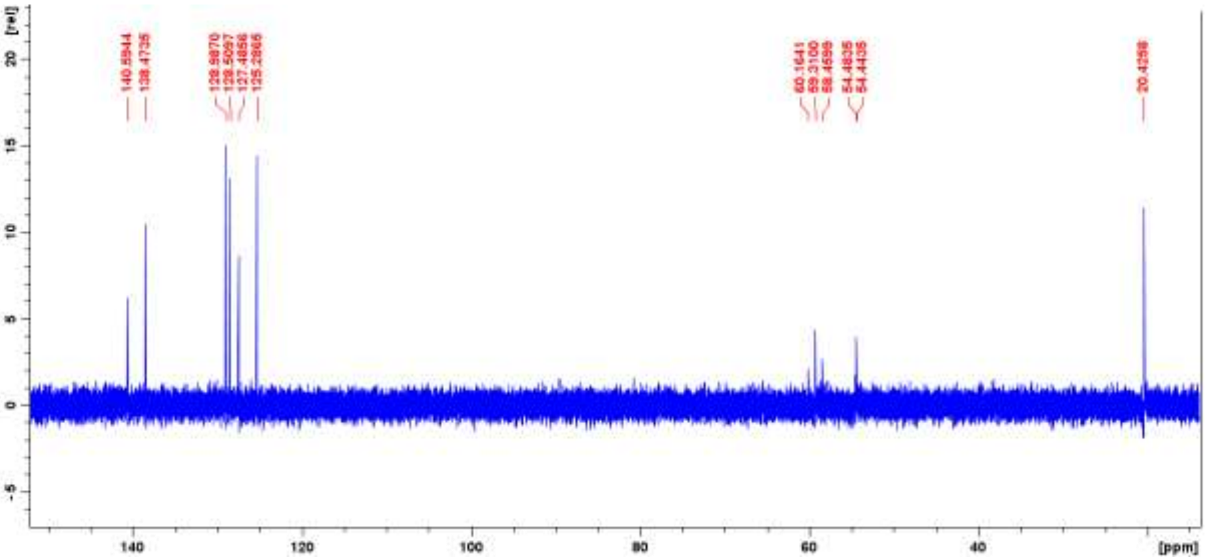

Figure S28. ^{13}C NMR spectrum of compounds **5b**

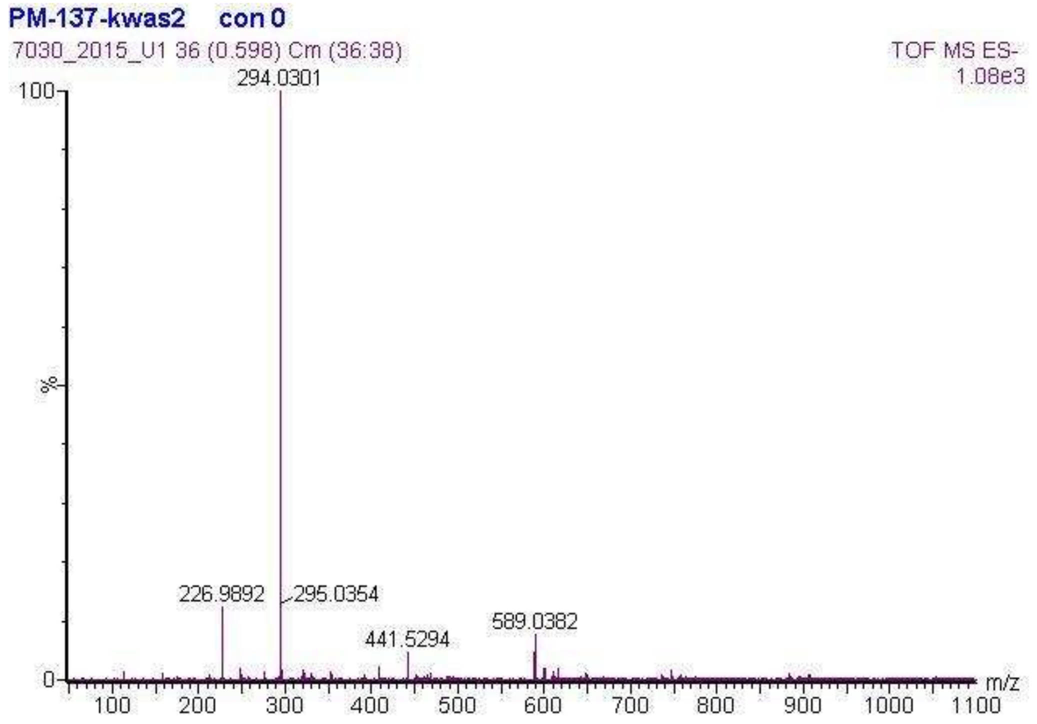

Figure S29. HRMS spectrum of compounds **5b**

compound **6b** 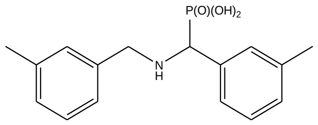

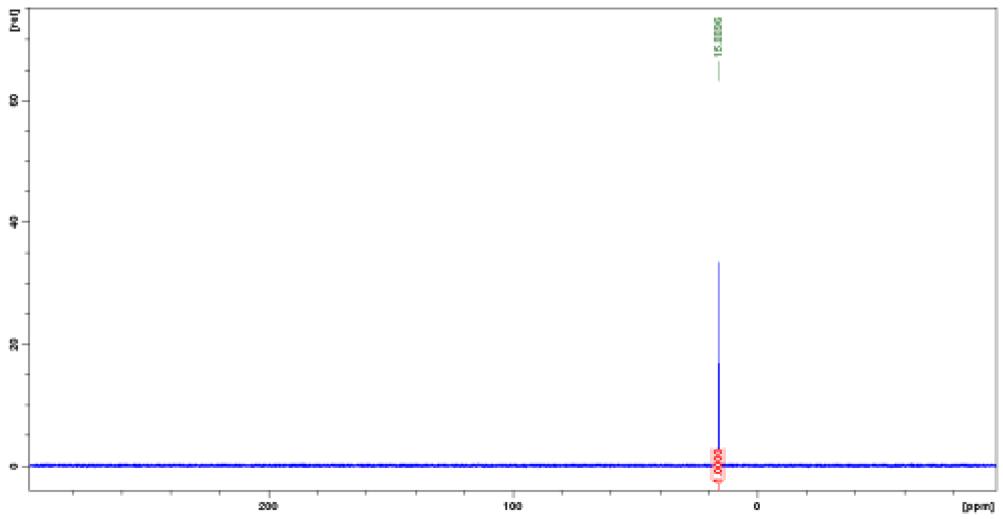

Figure S30. ^{31}P NMR spectrum of compounds **6b**

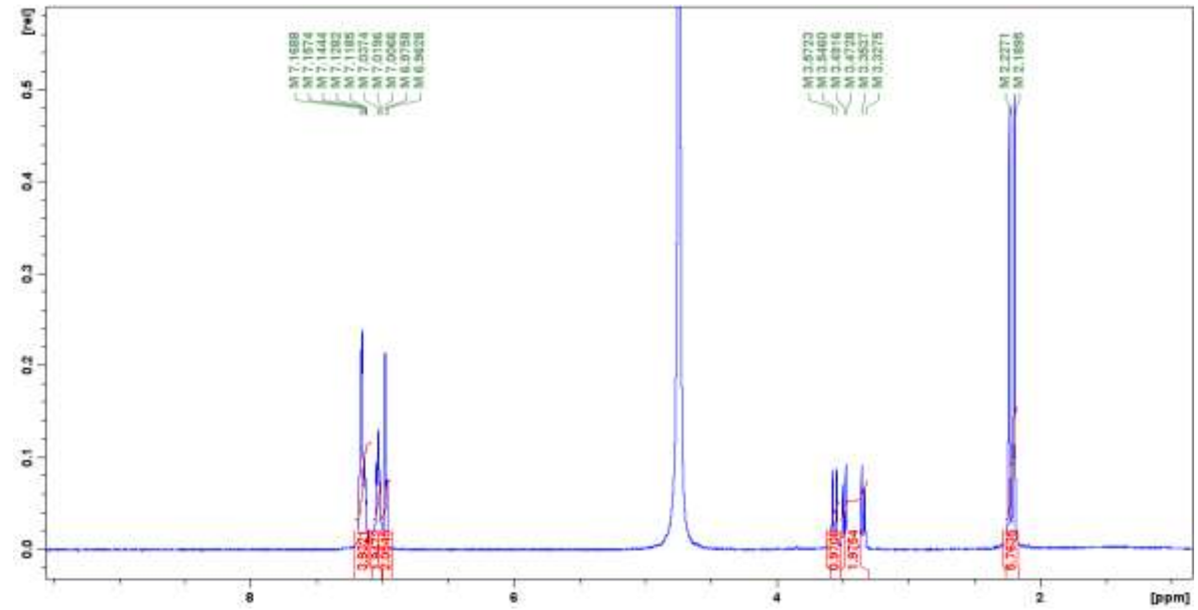

Figure S31. ^{1}H NMR spectrum of compounds **6b**

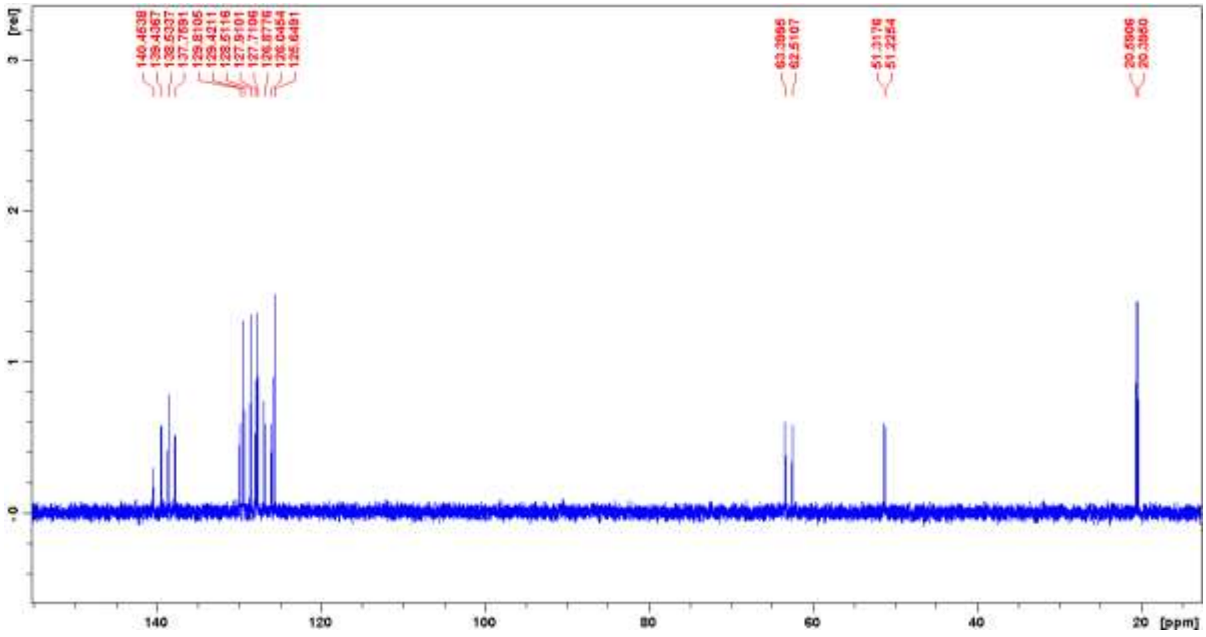

Figure S32. ^{13}C NMR spectrum of compounds **6b**

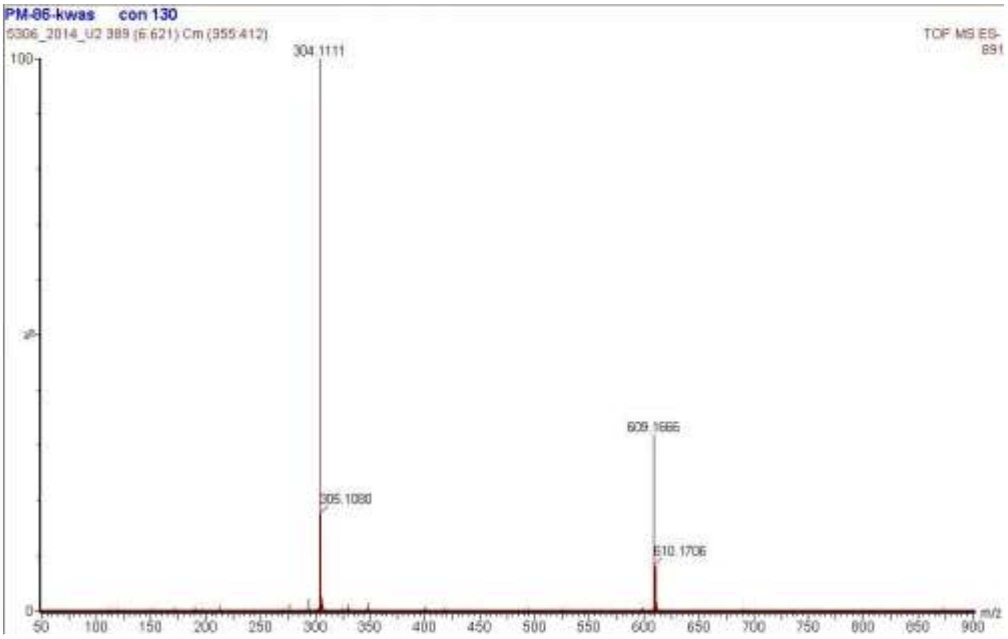

Figure S33. HRMS spectrum of compounds **6b**

compound **5c**

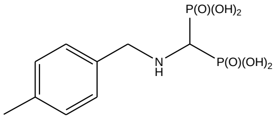

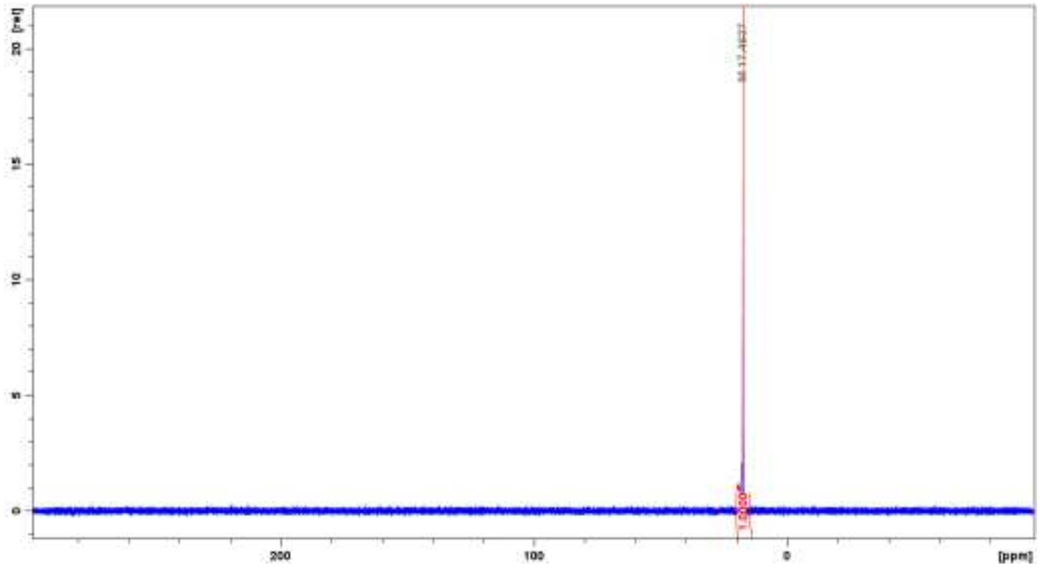

Figure S34. ^{31}P NMR spectrum of compounds **5c**

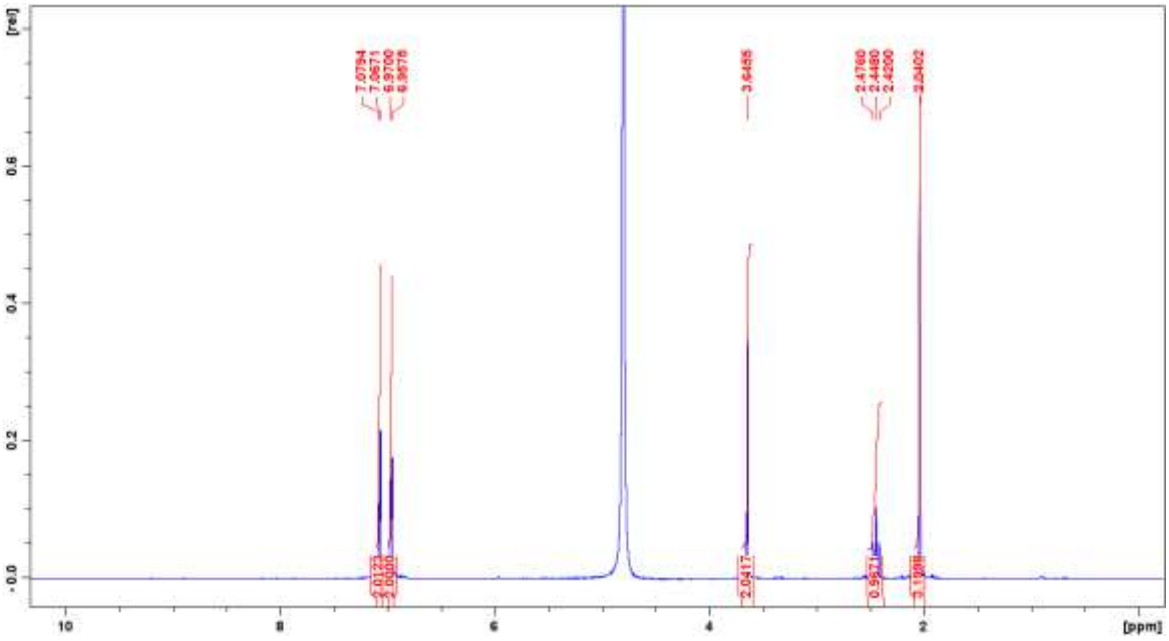

Figure S35. ^{1}H NMR spectrum of compounds **5c**

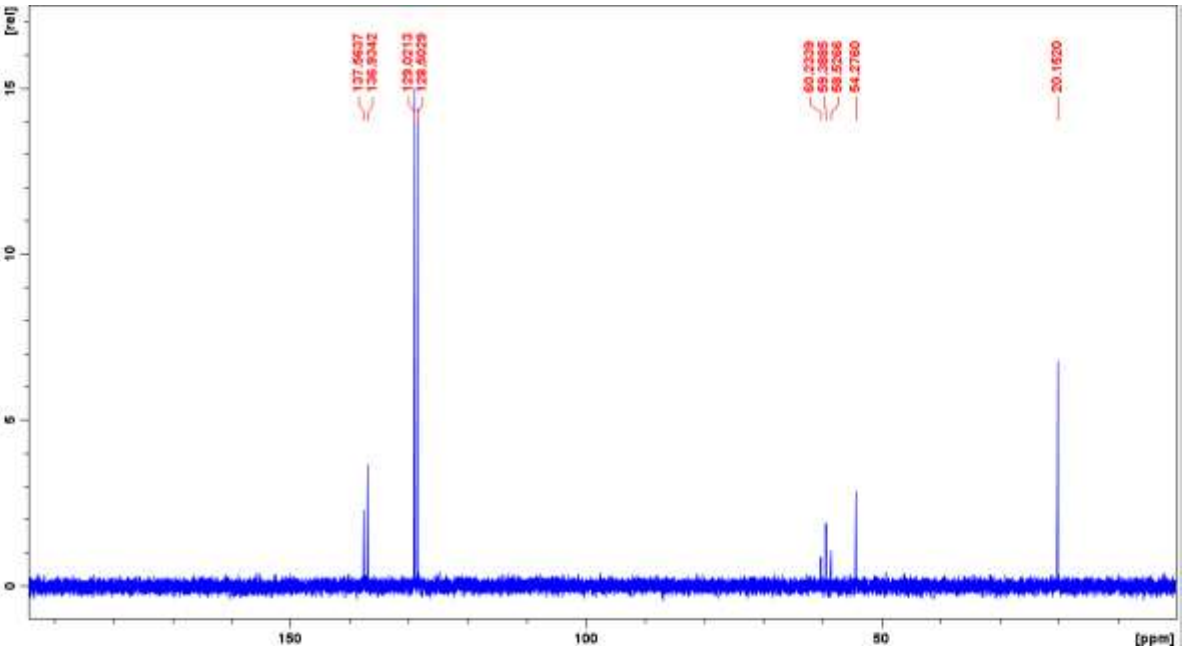

Figure S36. ^{13}C NMR spectrum of compounds **5c**

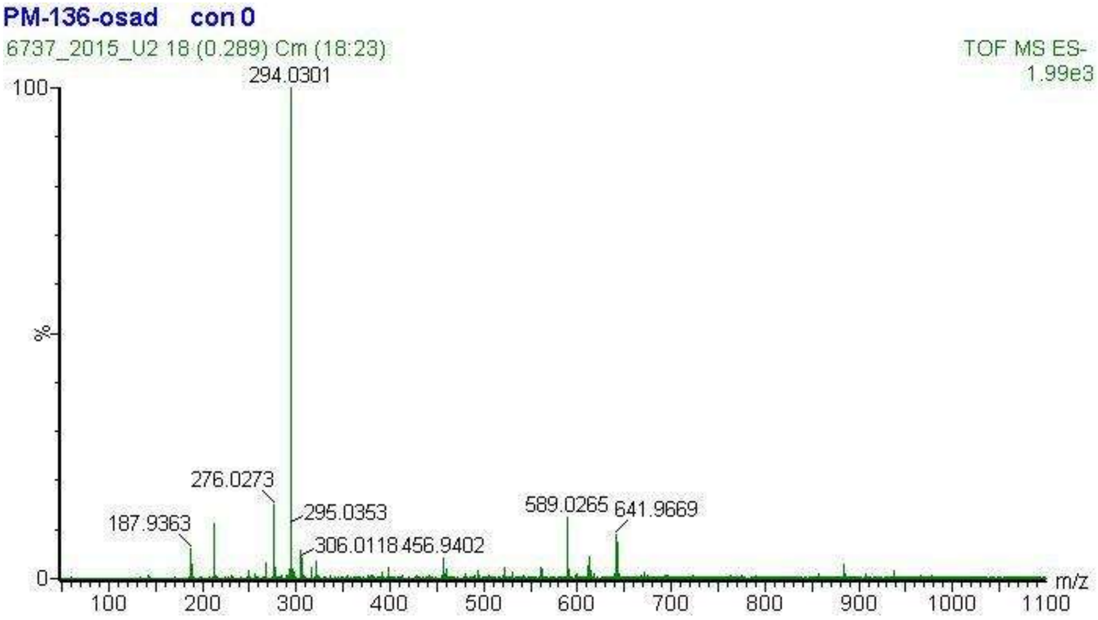

Figure S37. HRMS spectrum of compounds **5c**

compound **6c**

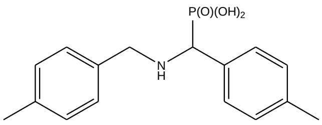

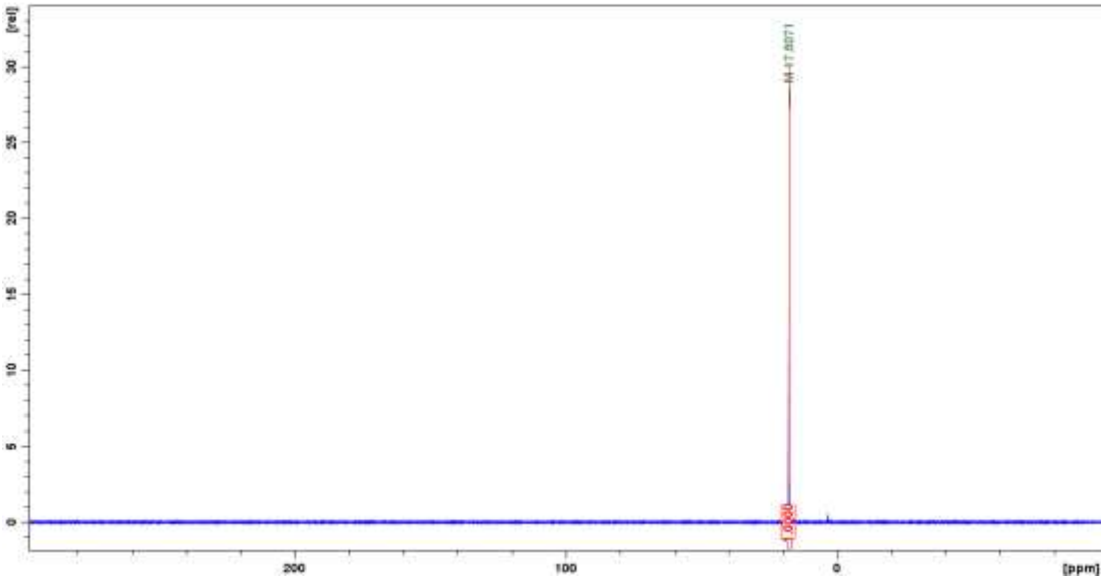

Figure S38. ^{31}P NMR spectrum of compounds **6c**

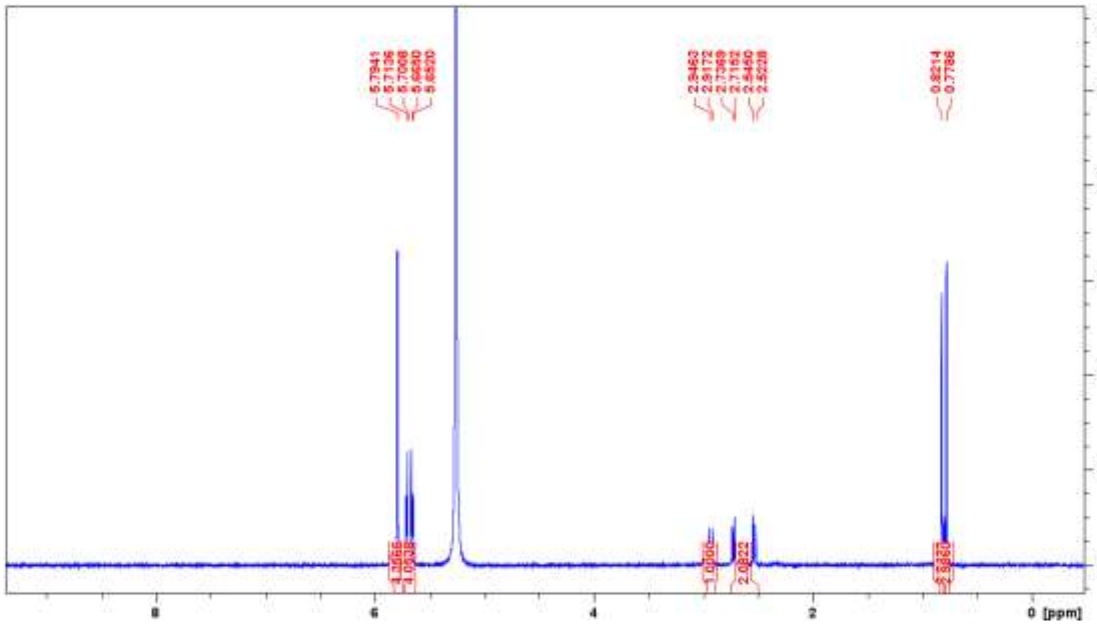

Figure S39. ^1H NMR spectrum of compounds **6c**

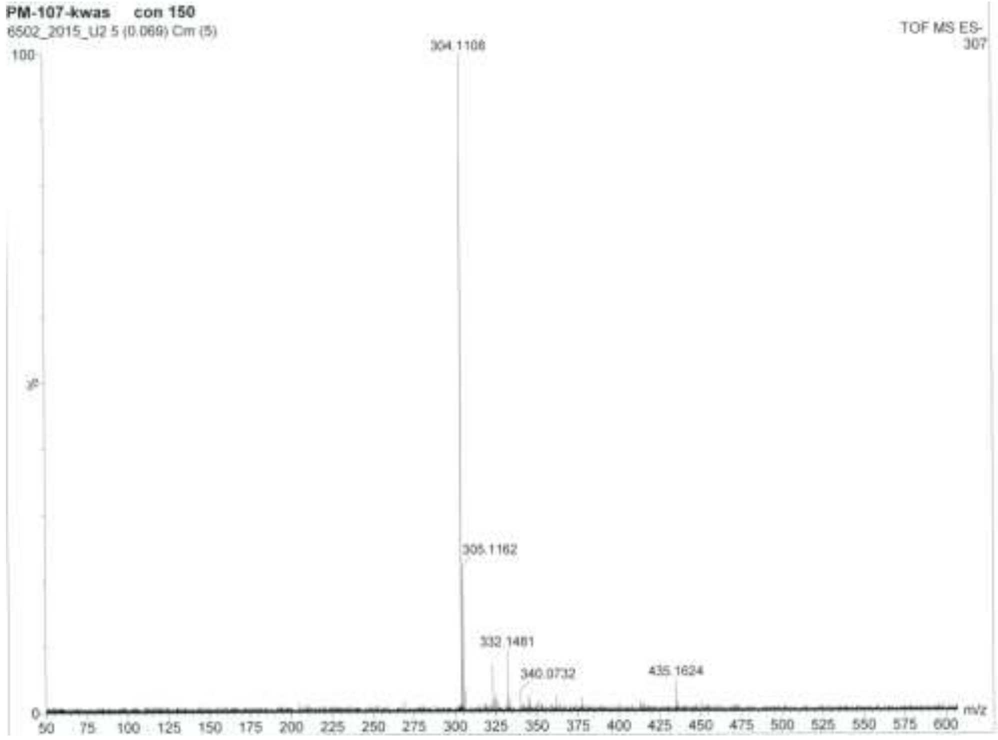

Figure S40. HRMS spectrum of compounds **6c**

Mixture of **5c** and **6c**

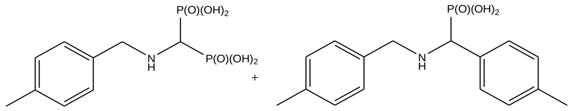

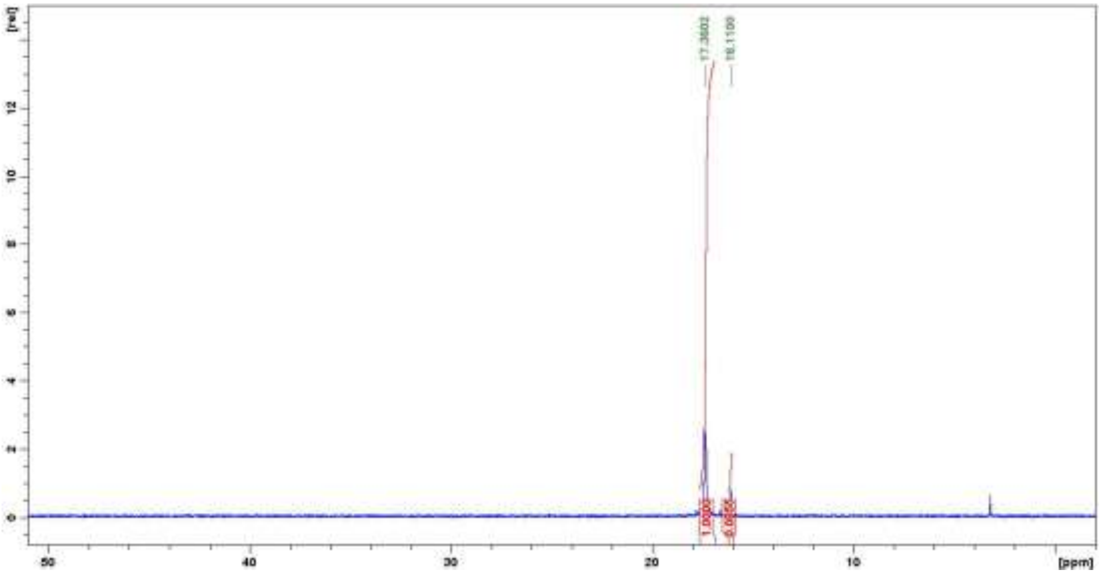

Figure S41. ^{31}P NMR spectrum of mixture of compounds **5c** and **6c**

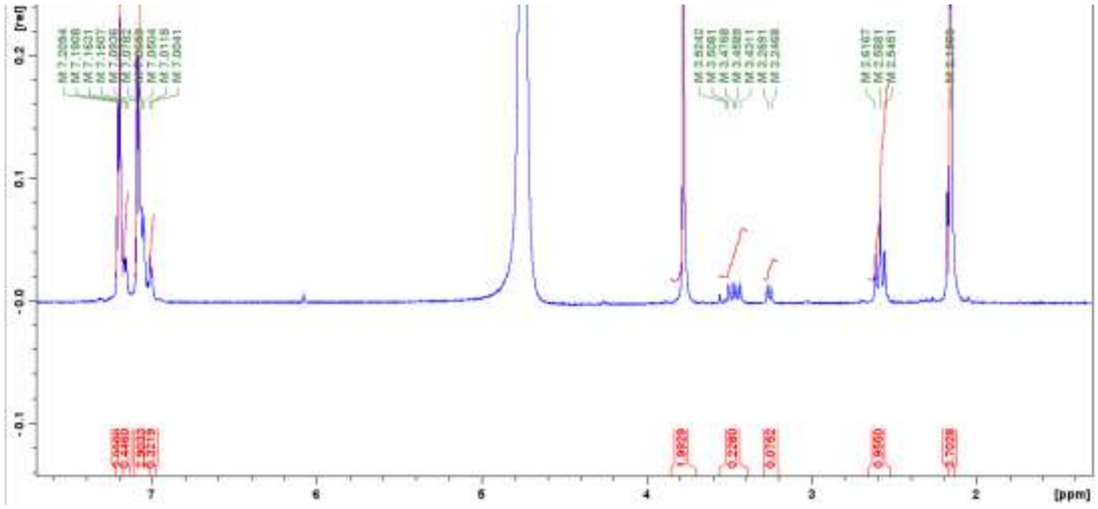

Figure S42. ^{1}H NMR spectrum of mixture of compounds **5c** and **6c**

compound **6d** 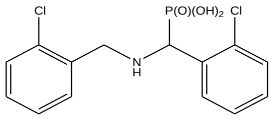

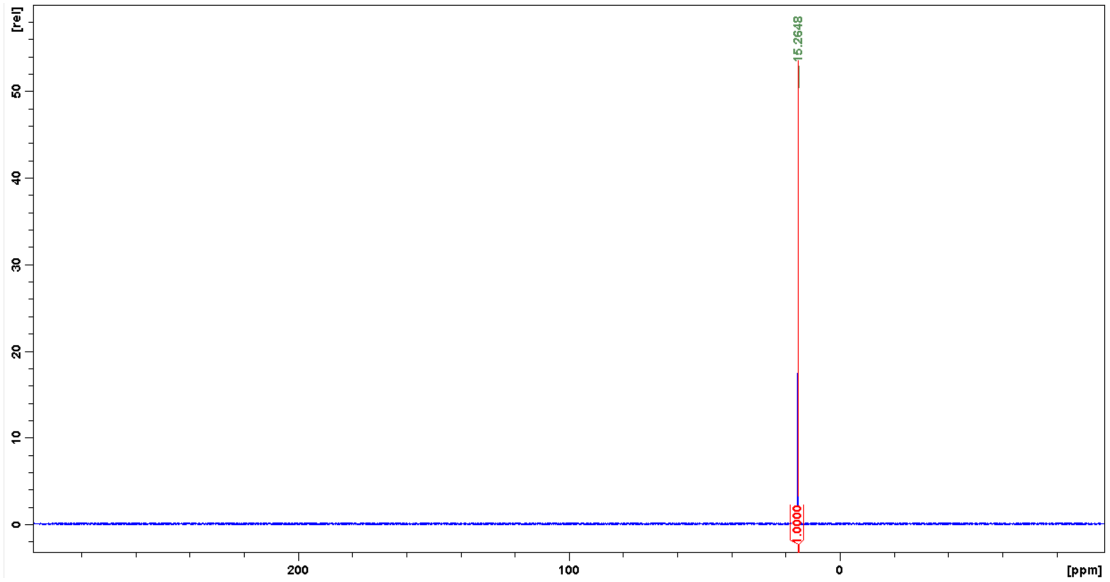

Figure S43. ^{31}P NMR spectrum of compounds **6d**

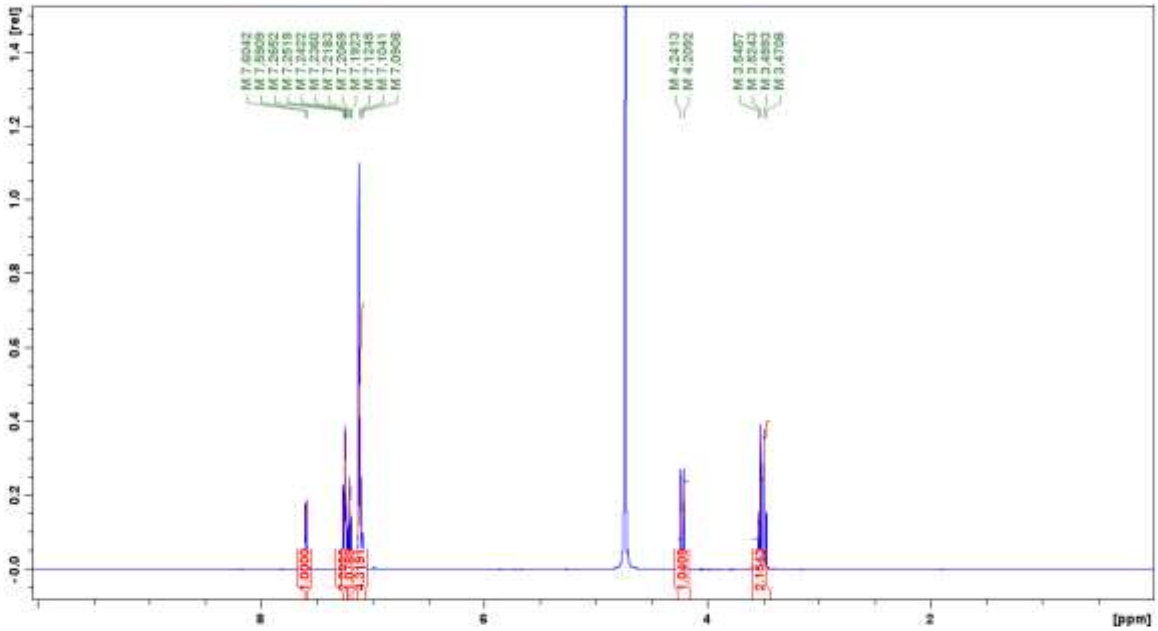

Figure S44. ^{1}H NMR spectrum of compounds **6d**

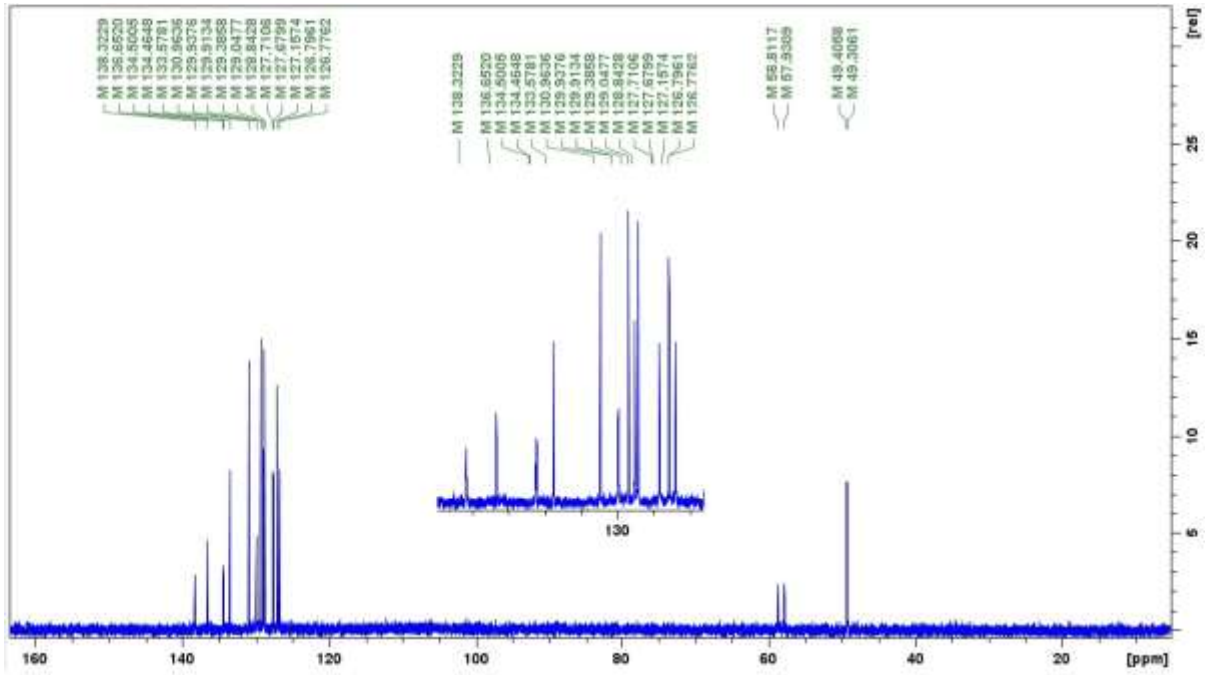

Figure S45. ^{13}C NMR spectrum of compounds **6d**

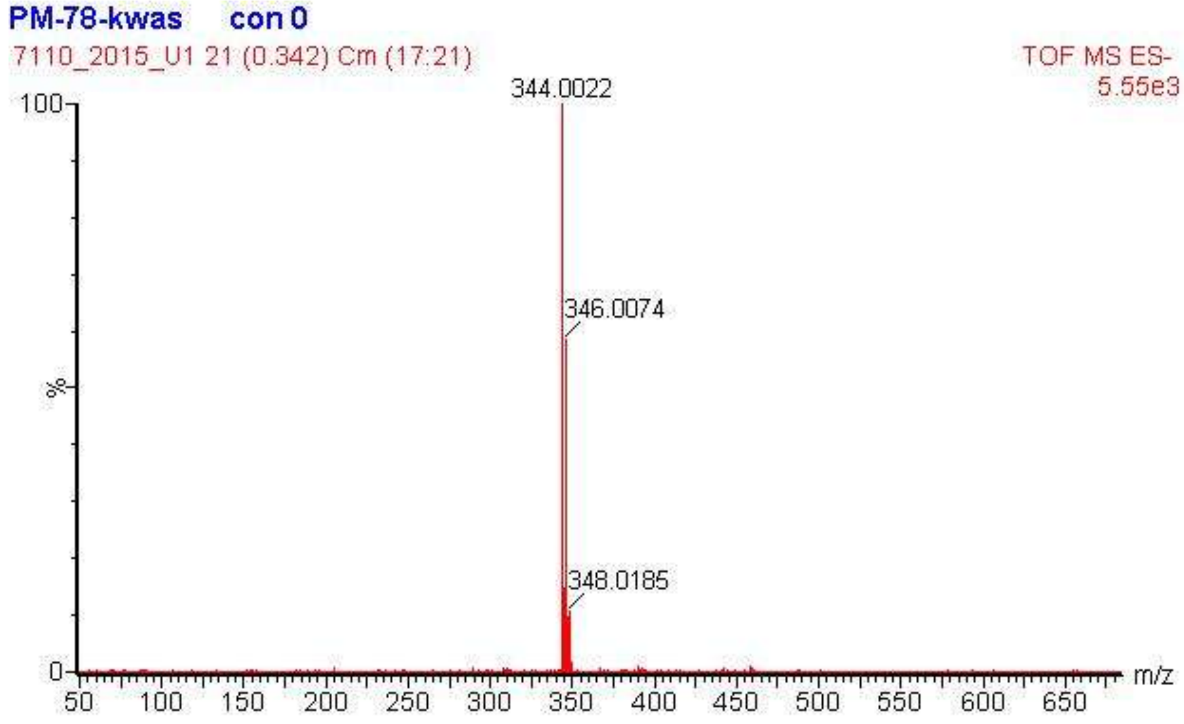

Figure S46. HRMS spectrum of compounds **6d**

compound **6e**

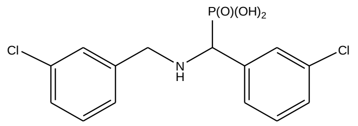

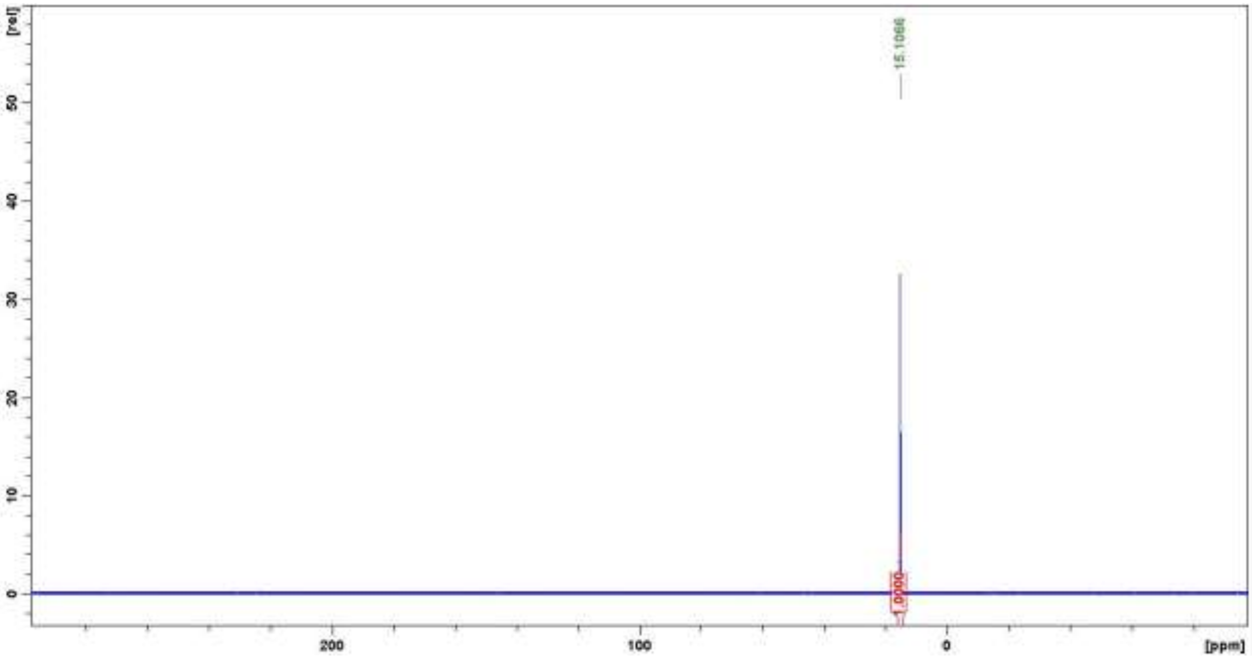

Figure S47. ^{31}P NMR spectrum of compounds **6e**

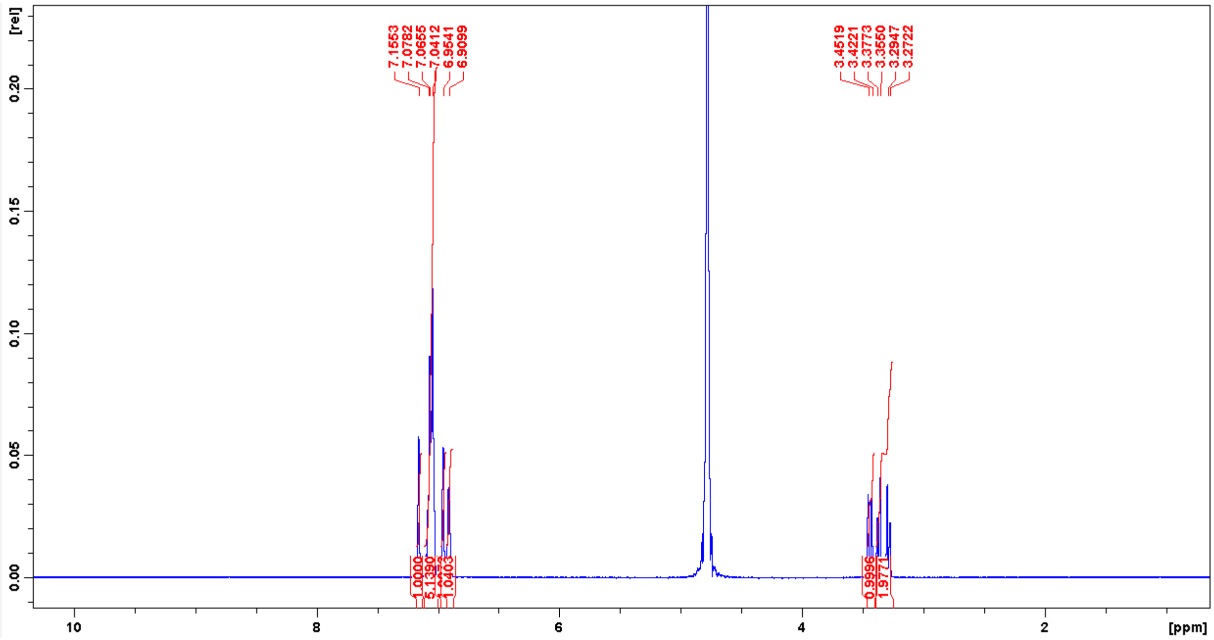

Figure S48. ^{1}H NMR spectrum of compounds **6e**

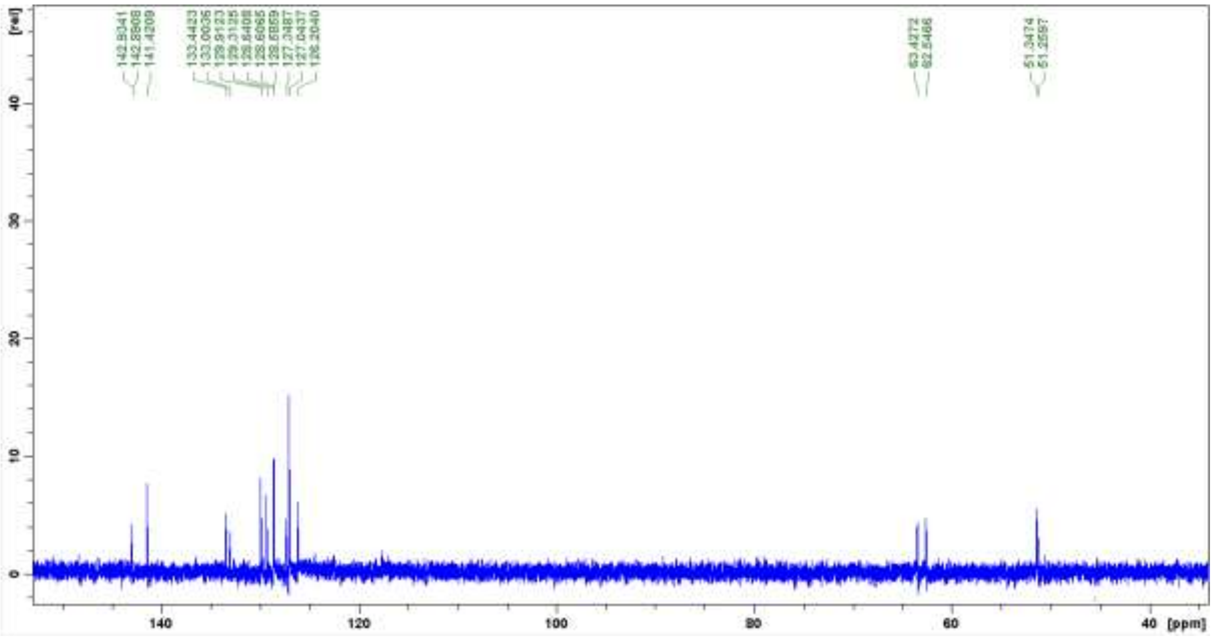

Figure S49. ^{13}C NMR spectrum of compounds **6e**

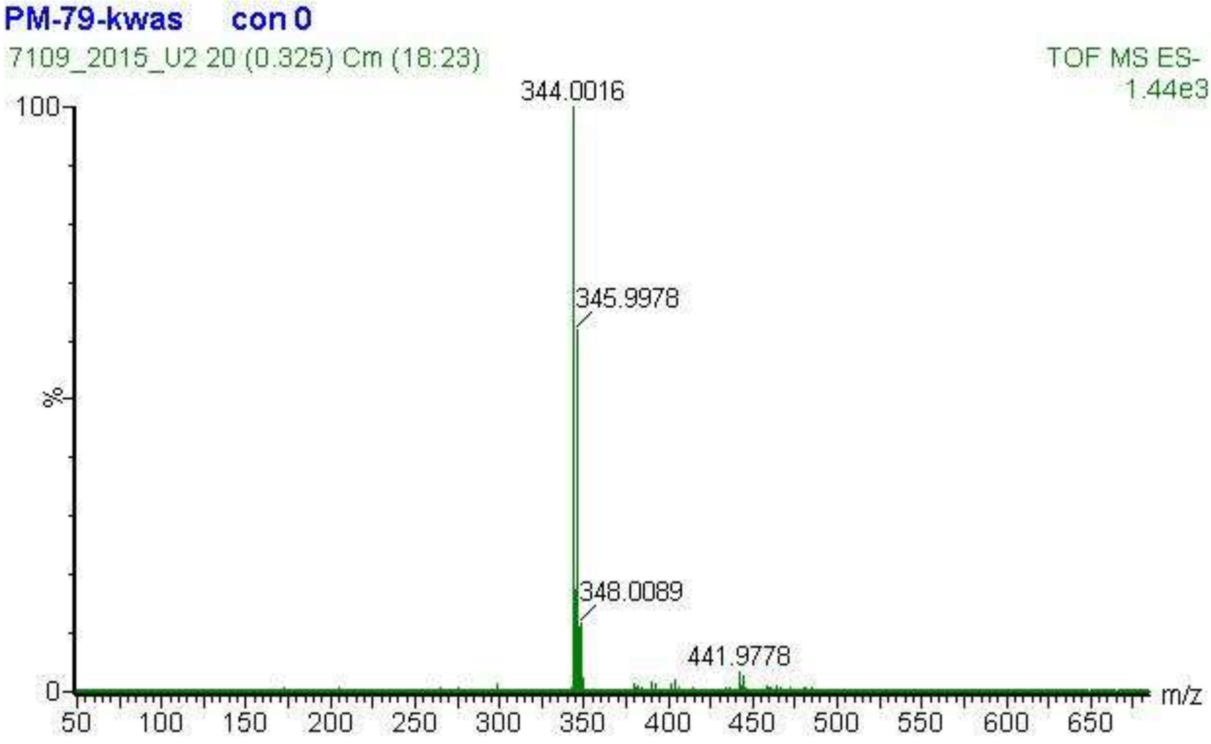

Figure S50. HRMS spectrum of compounds **6e**

mixture of compounds **5g+6g**

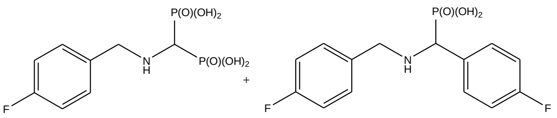

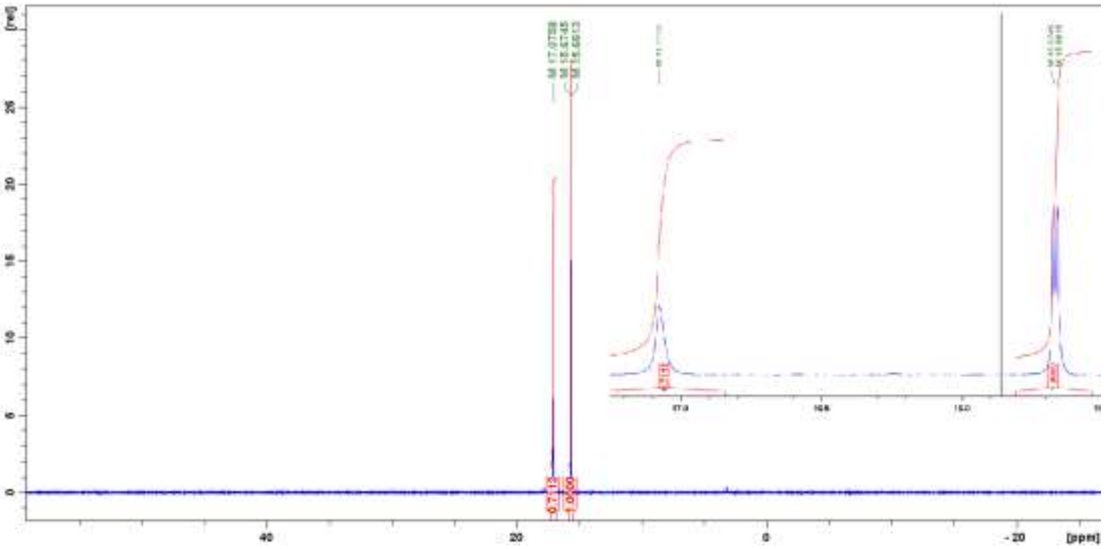

Figure S51.^{31}P NMR spectrum of mixture of compounds **5g** and **6g**

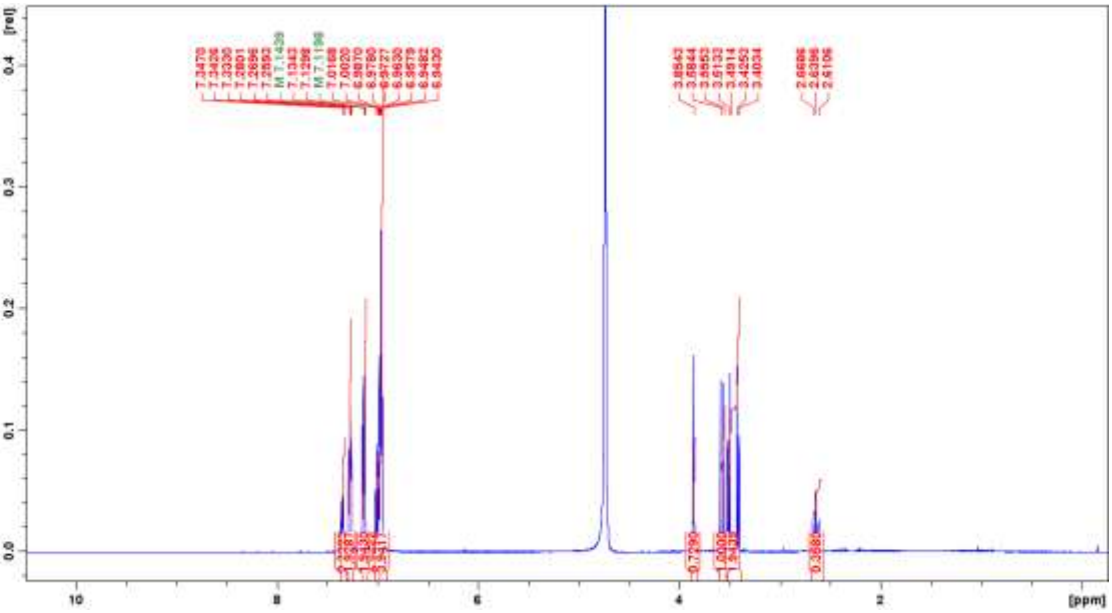

Figure S52. ^{1}H NMR spectrum of mixture of compounds **5g** and **6g**

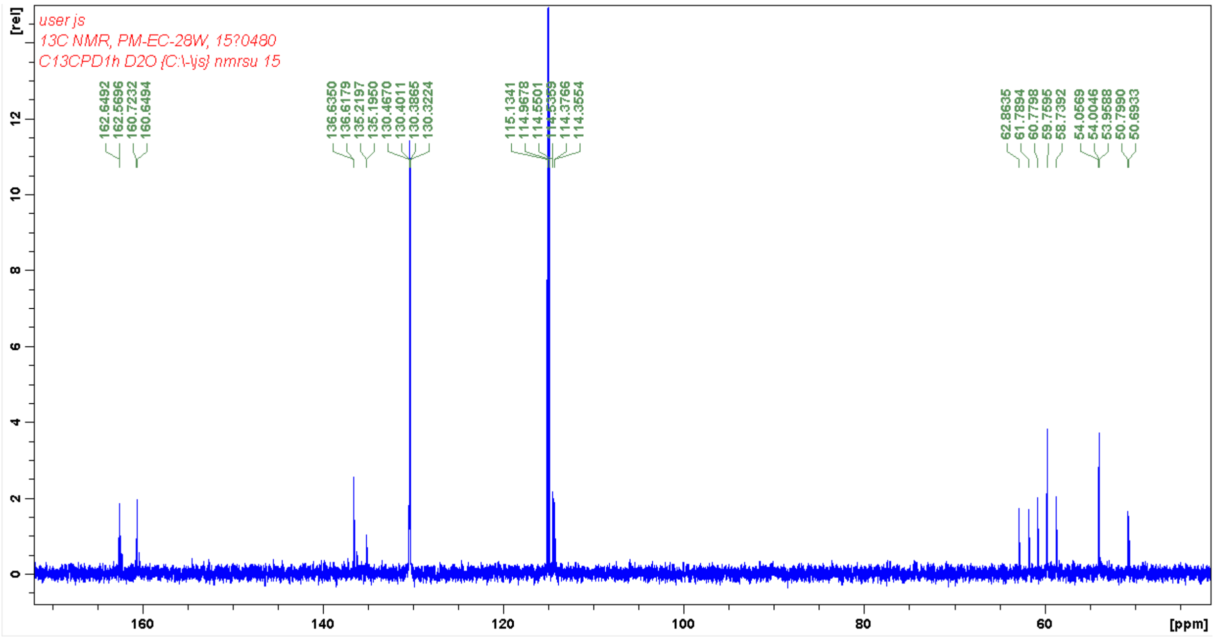

Figure S53. ^{13}C NMR spectrum of mixture of compounds **5g** and **6g**

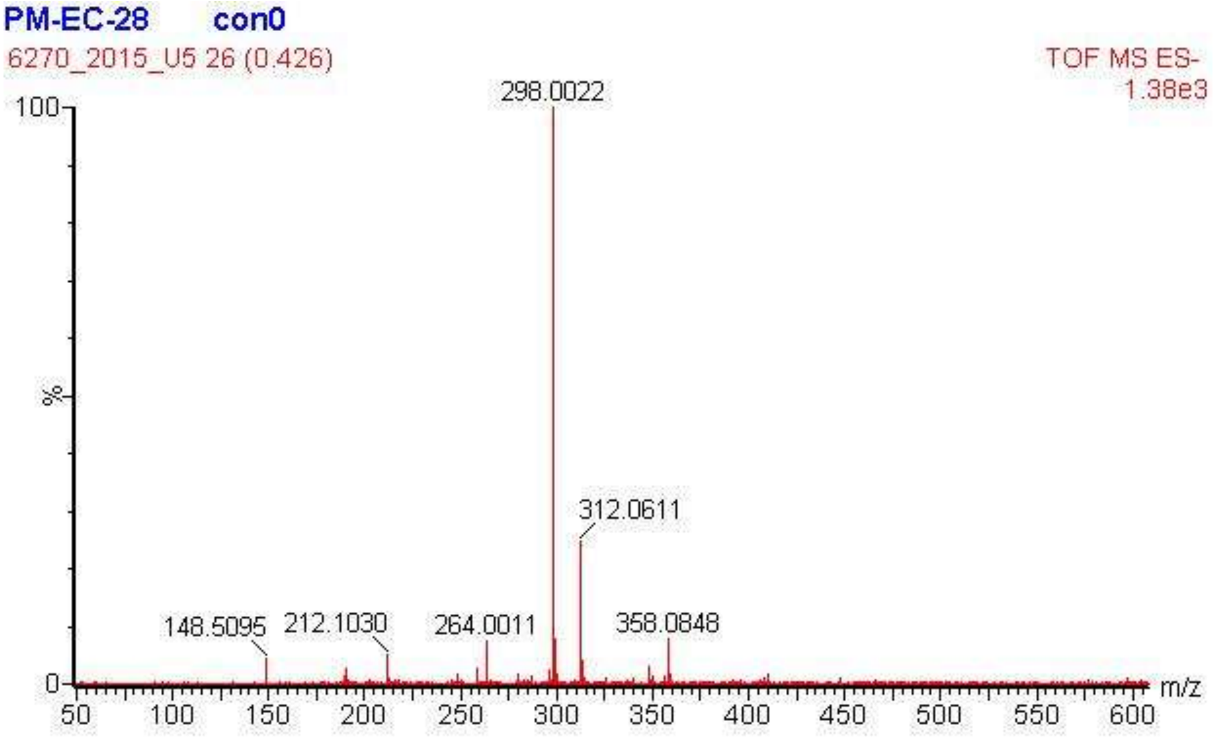

Figure S54. HRMS spectrum of mixture of compounds **5g** and **6g**

compound **5h** 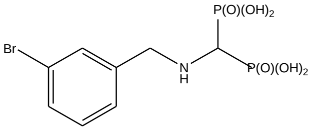

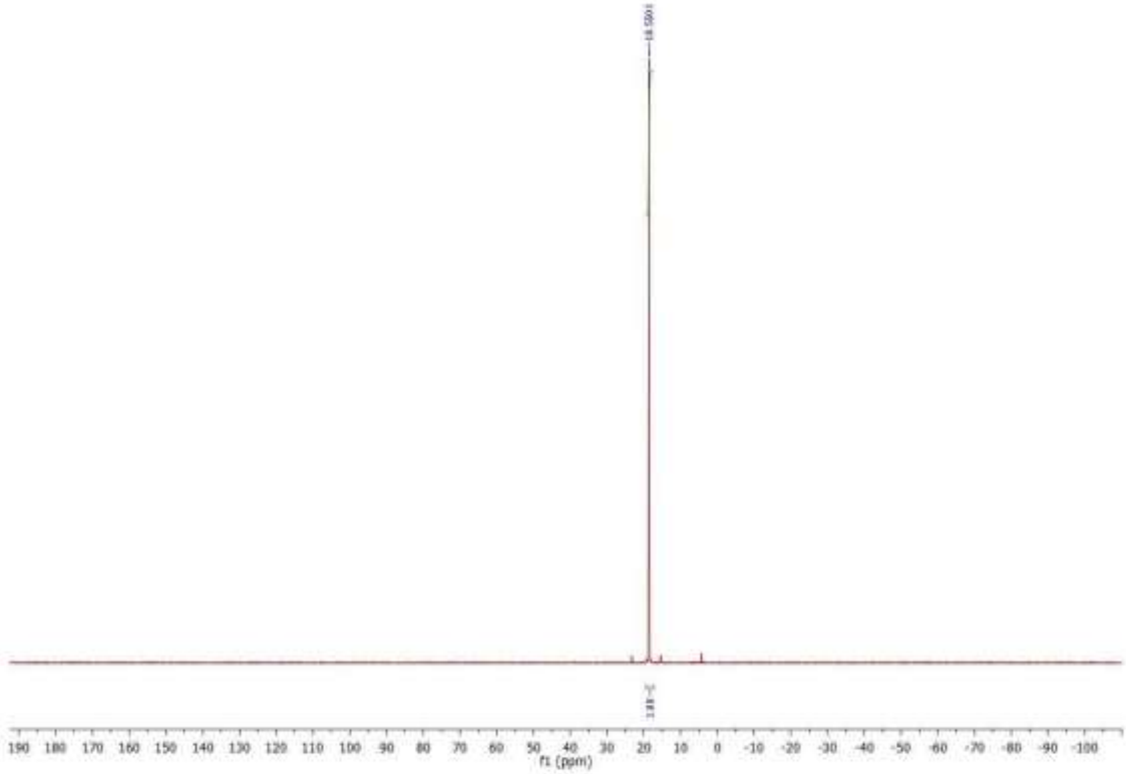

Figure S55. ^{31}P NMR spectrum of compound **5h**

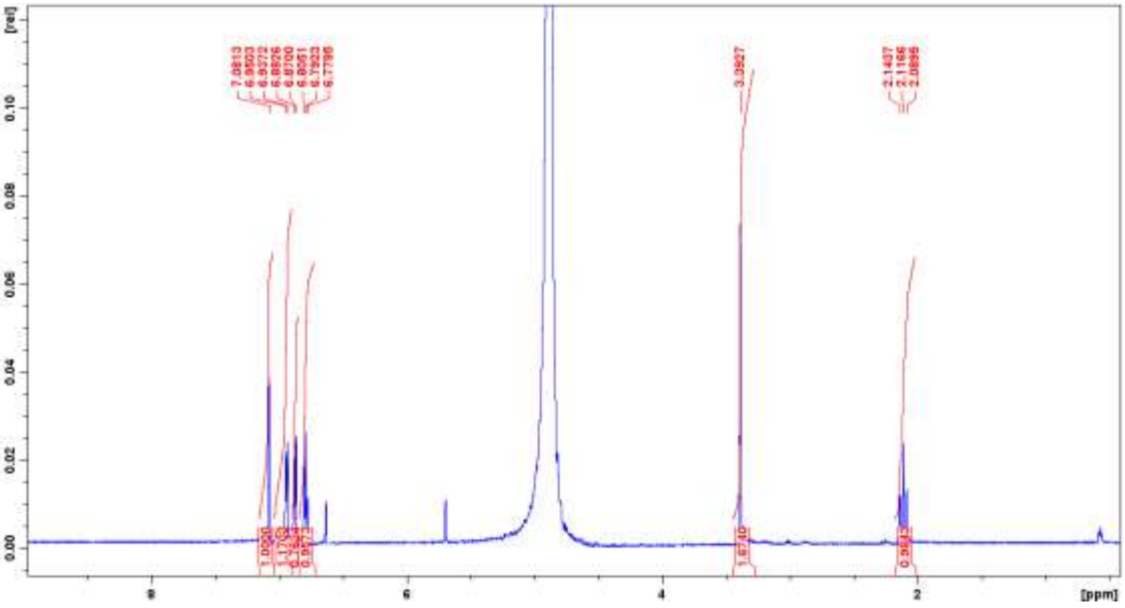

Figure S56. ^{1}H NMR spectrum of compound **5h**

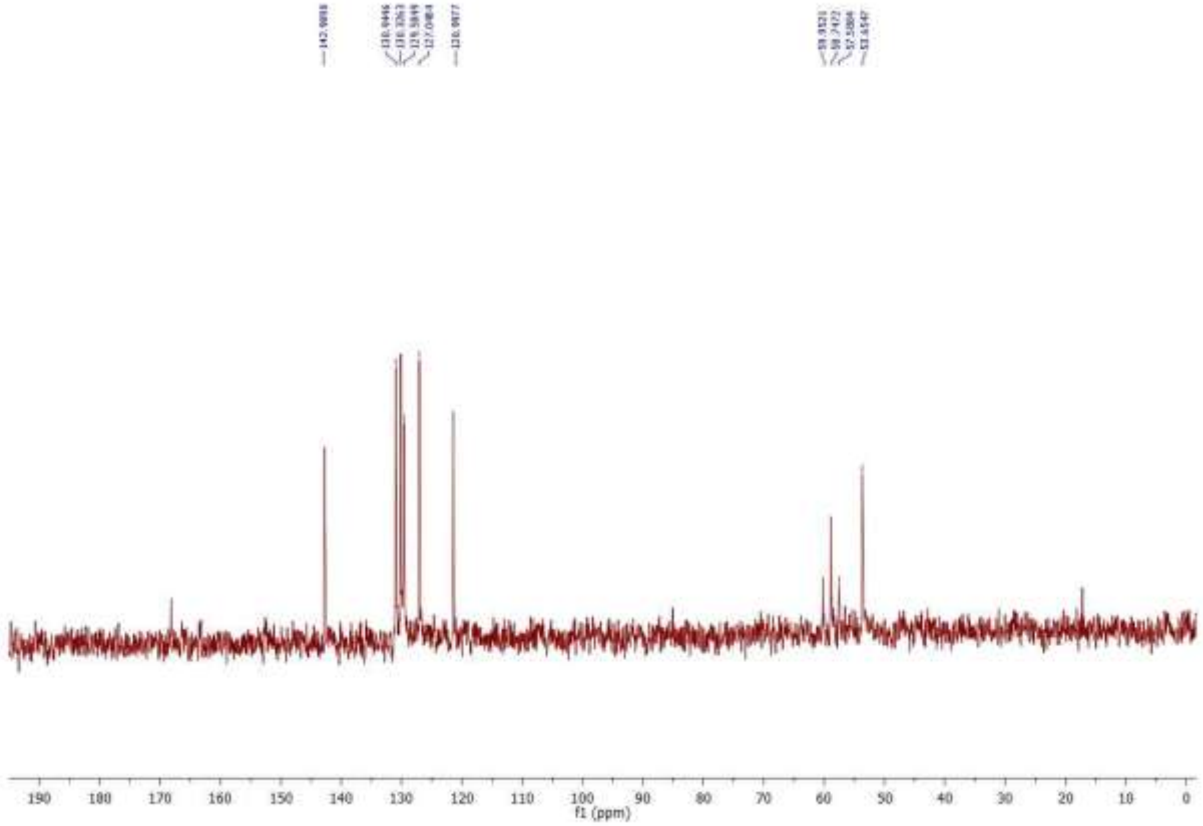

Figure S57. ^{13}C NMR spectrum of compound **5h**

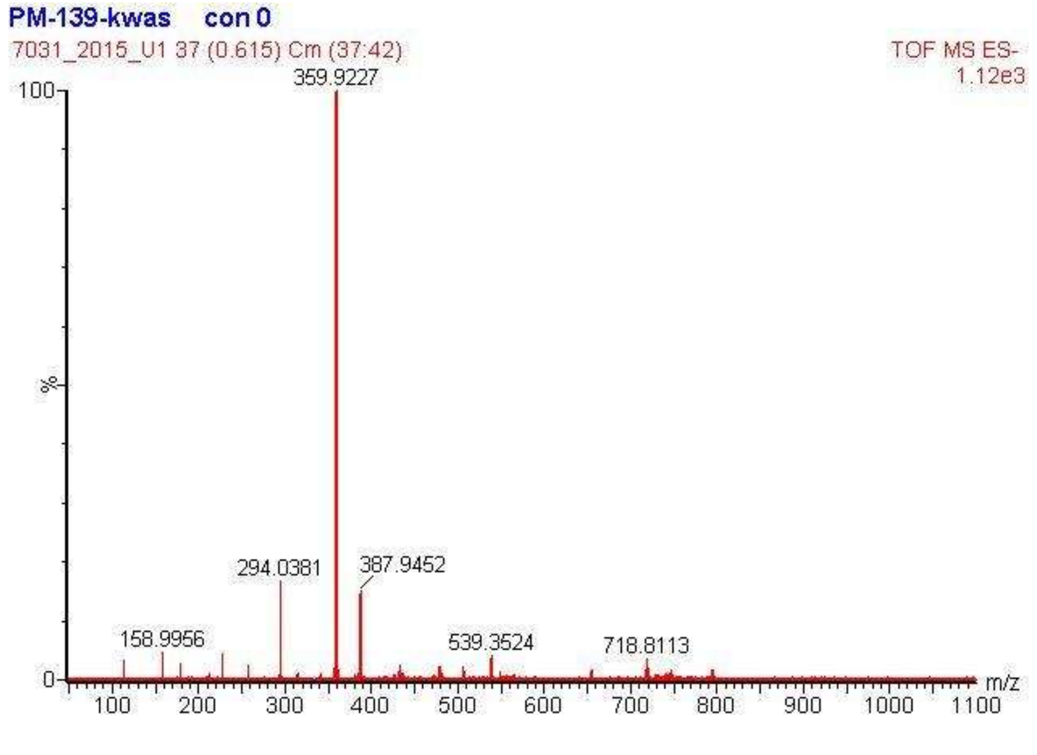

Figure S58. HRMS spectrum of compound **5h**

compound **15** 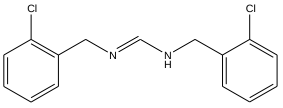

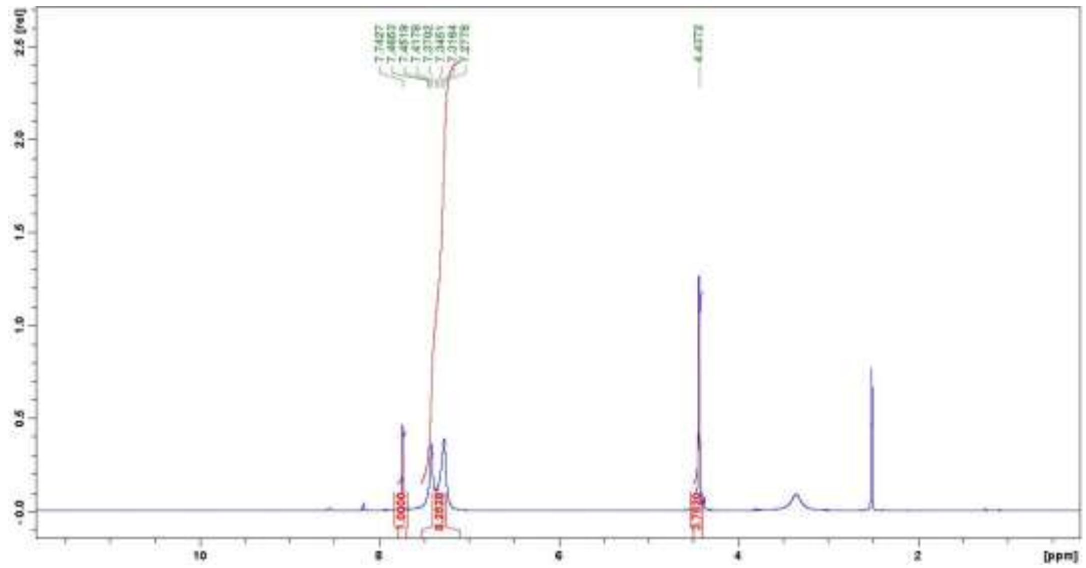

Figure S59. ^{1}H NMR spectrum of compound **15**

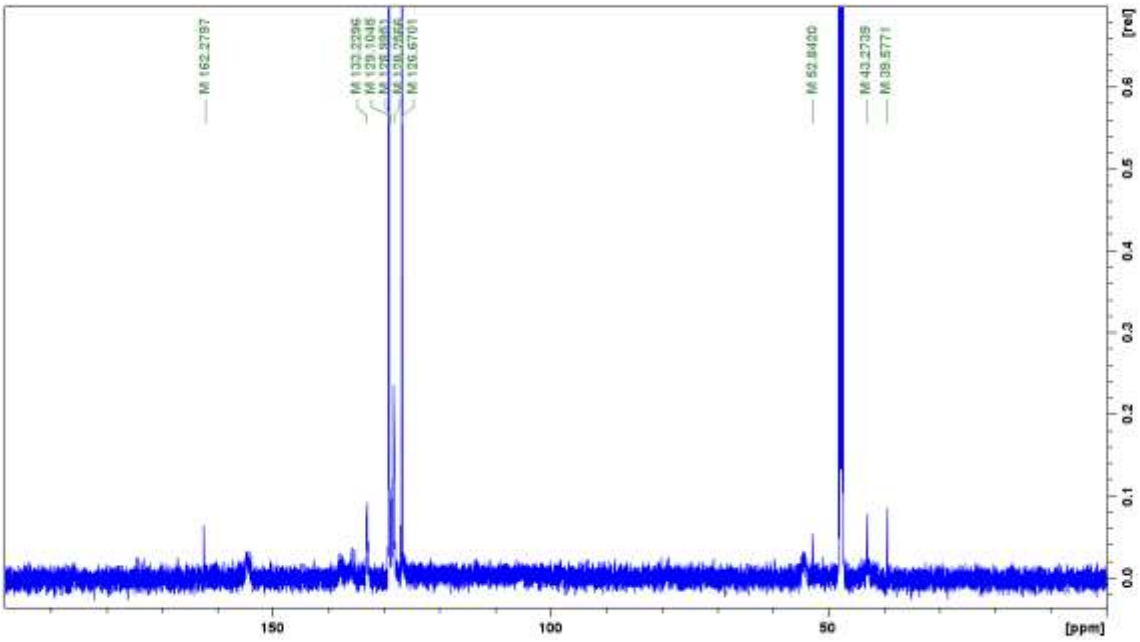

Figure S60. ^{13}C NMR spectrum of compound **15**

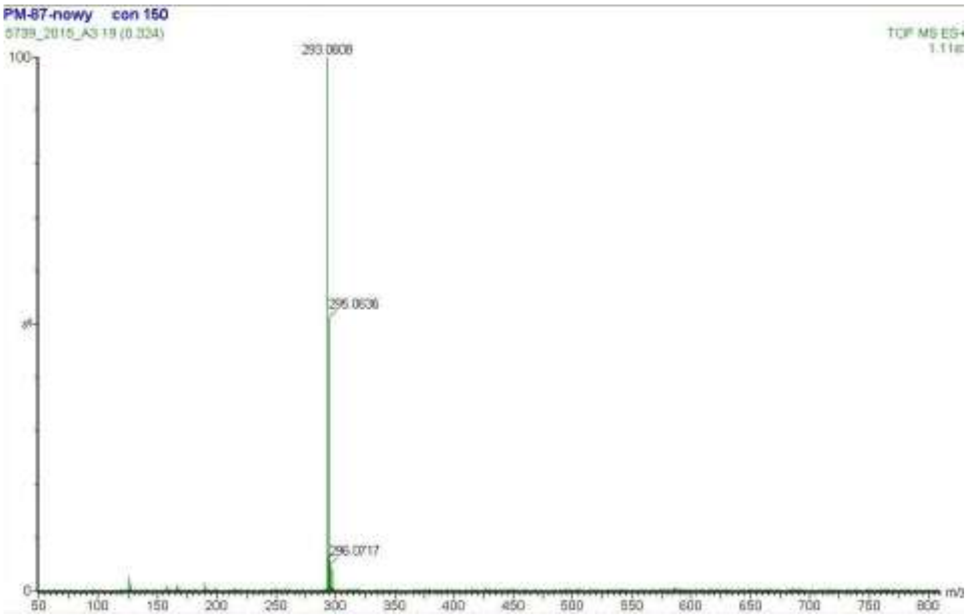

Figure S61. [1]HRMS spectrum of compound **15**

*mixture of compounds **6d** and **16***

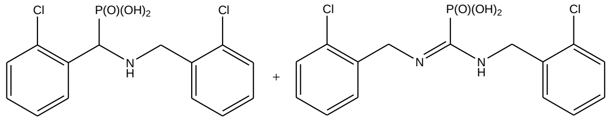

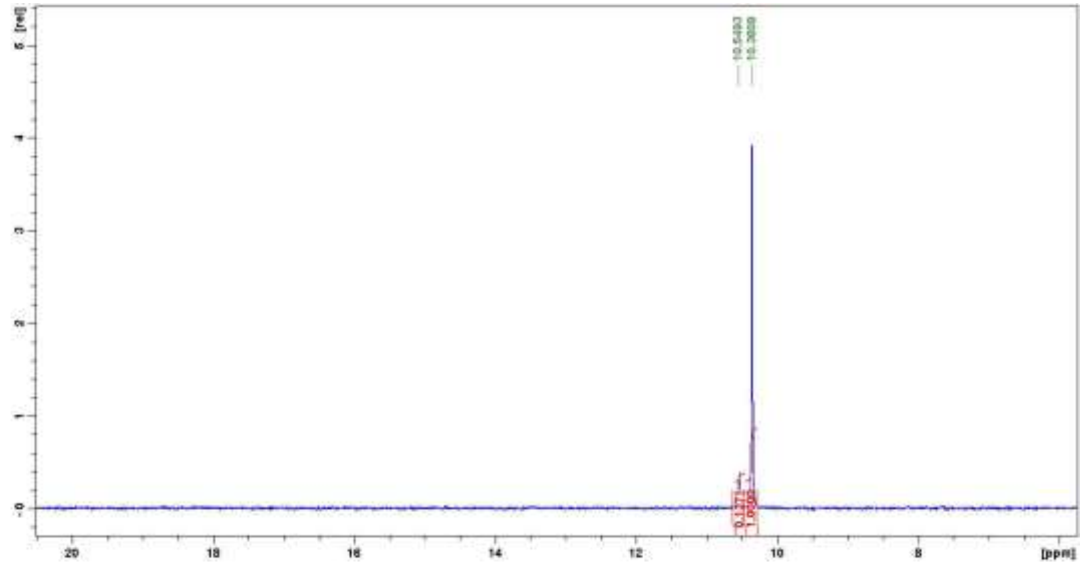

Figure S62. ^{31}P NMR spectrum of *mixture of compounds **6d** and **16***

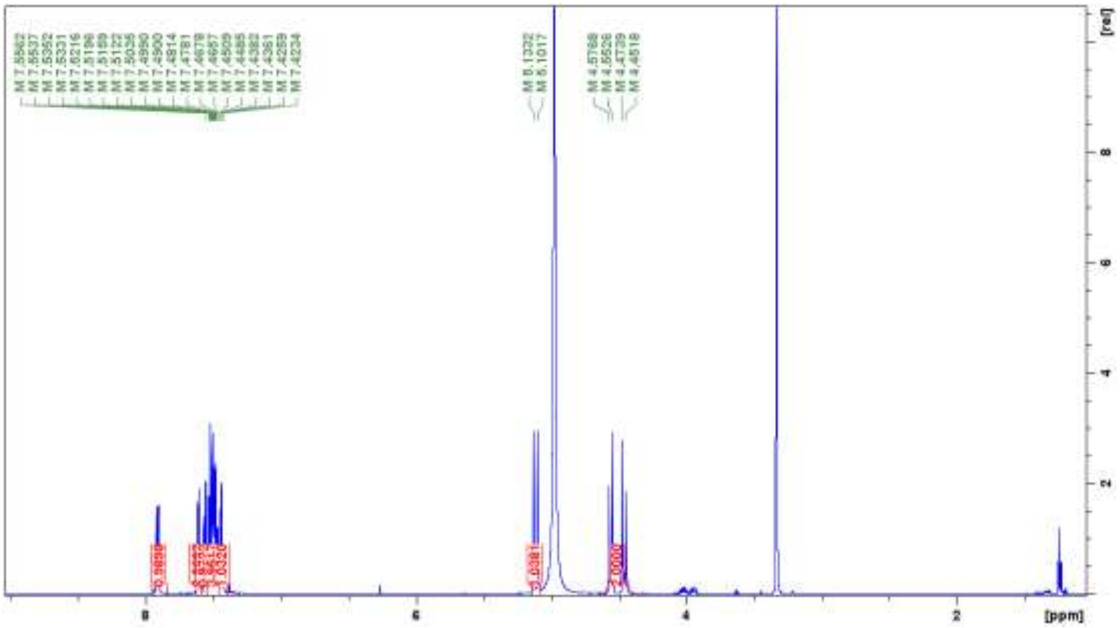

Figure S63. ^{1}H NMR spectrum of *mixture of compounds **6d** and **16***

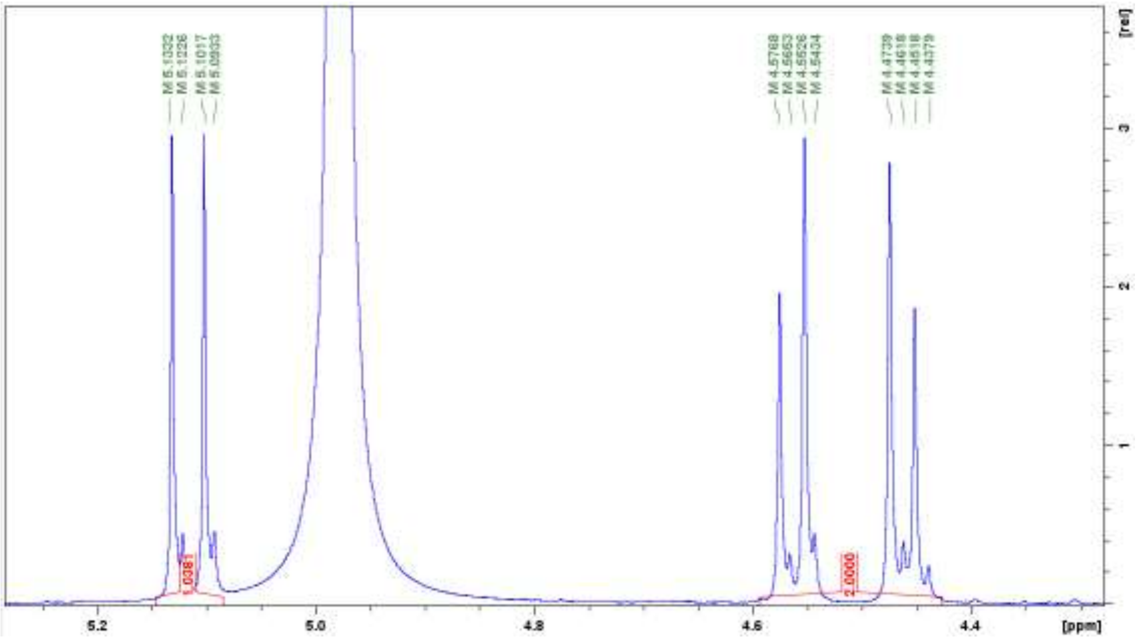

Figure S64. ^{1}H NMR spectrum of *mixture of compounds **6d** and **16** – wider range*

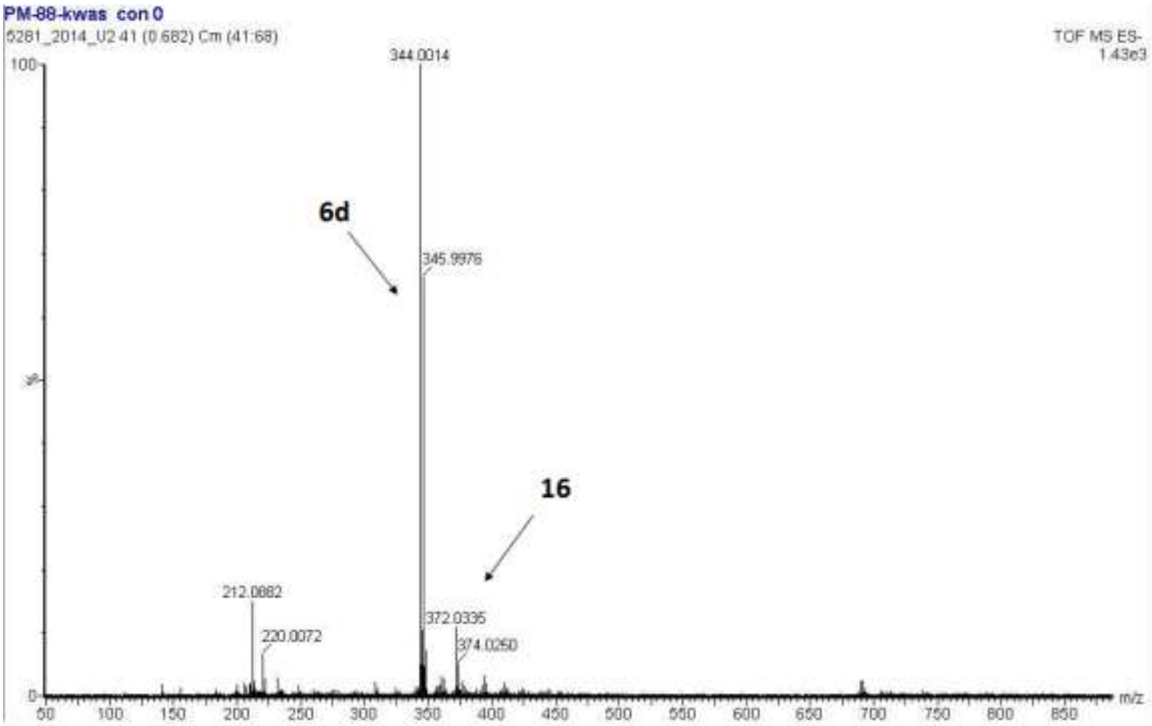

Figur

S65. HRMS spectrum of *mixture of compounds **6d** and **16***

compound ***8***

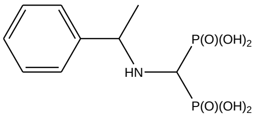

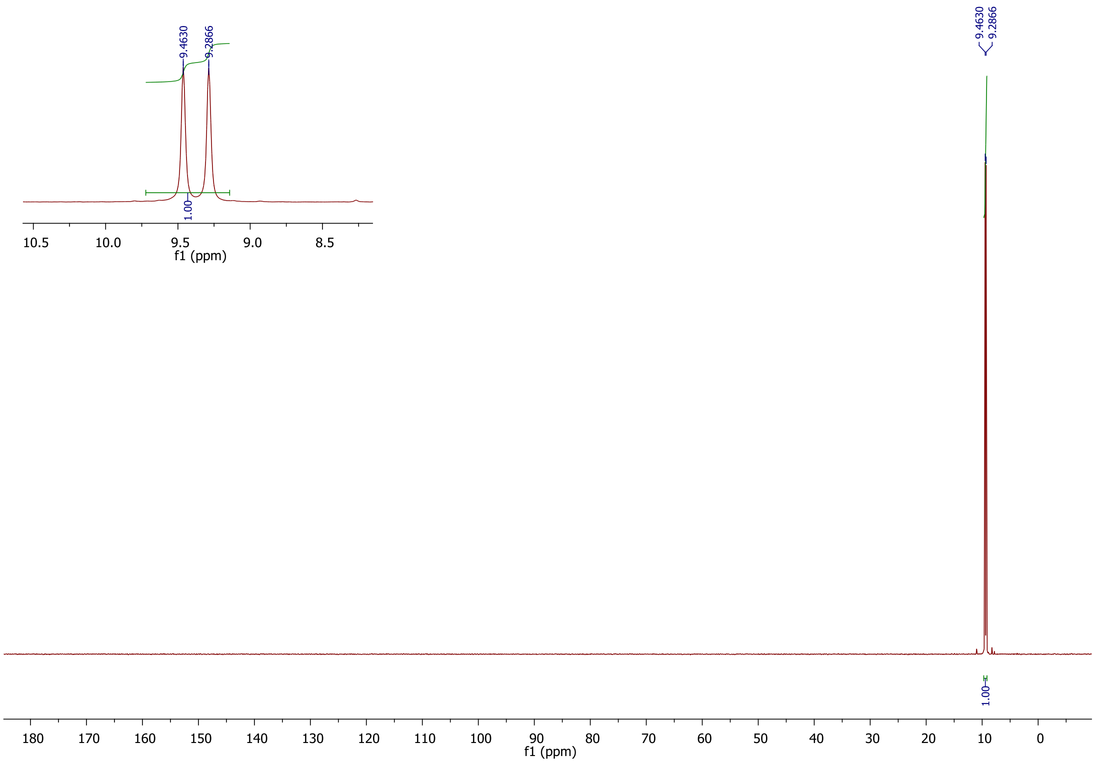

Figure S66. ^{31}P NMR spectrum of compound **8**

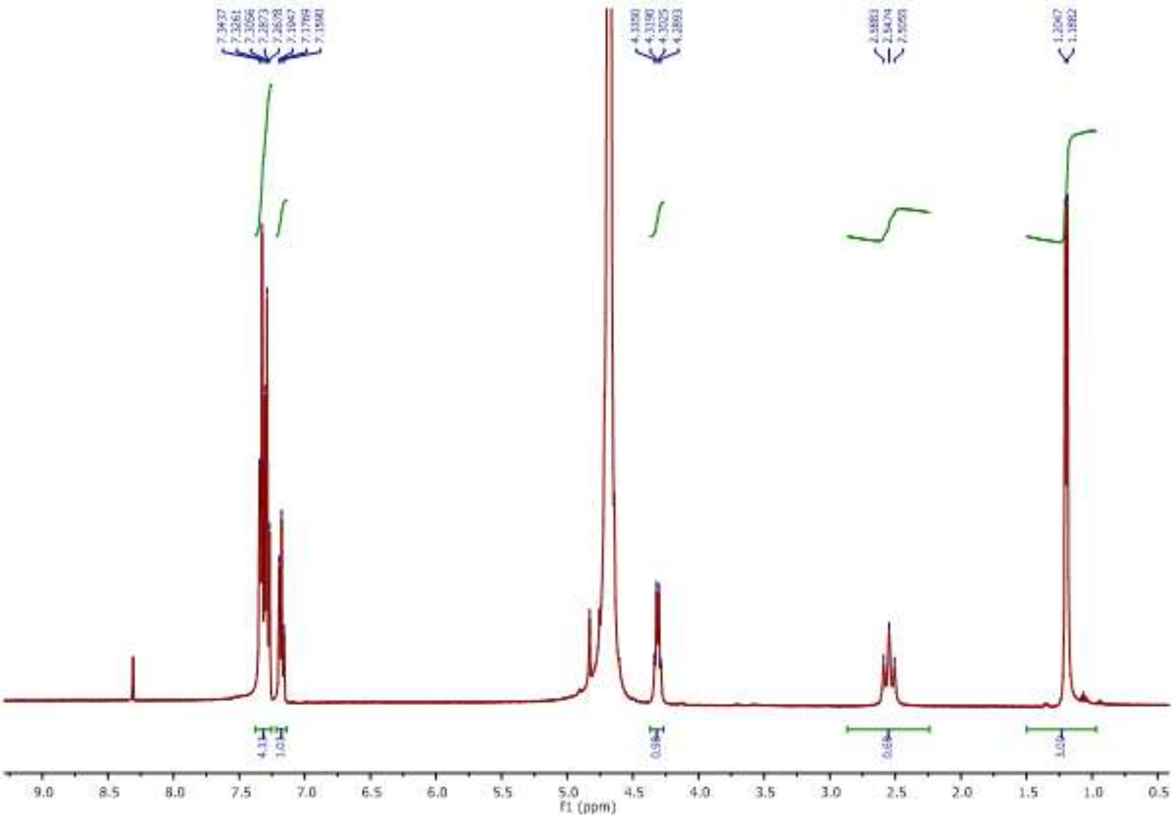

Figure S67. ^{1}H NMR spectrum of compound **8**

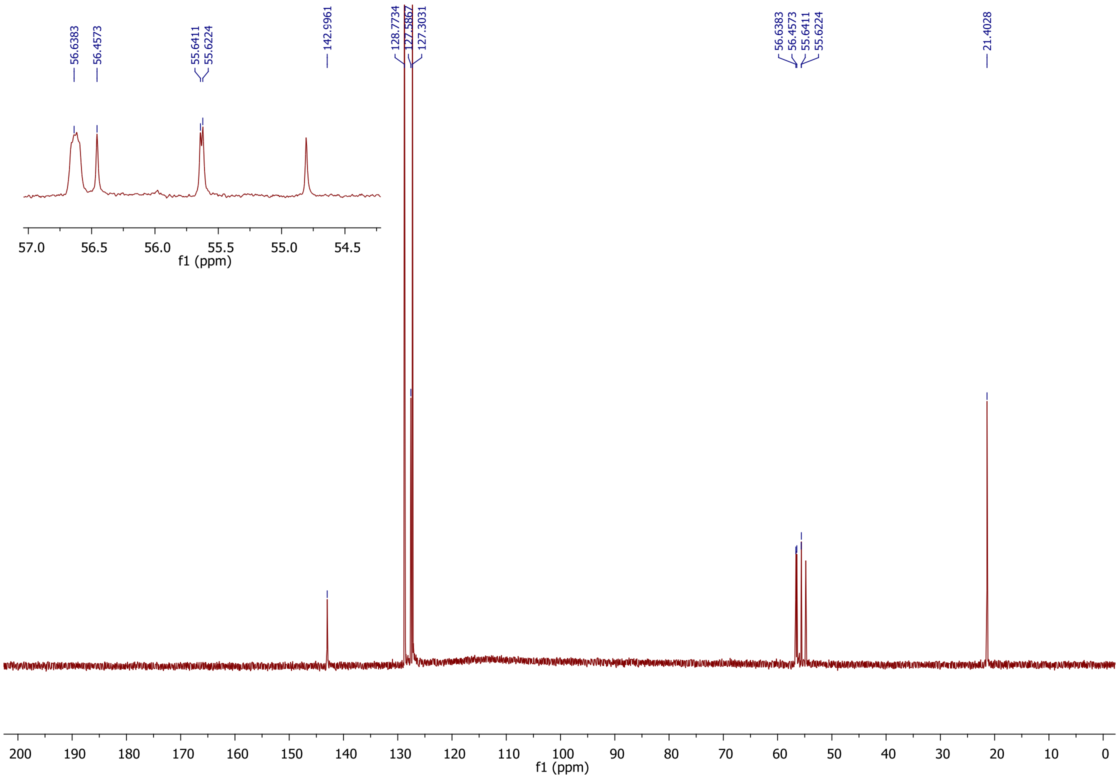

Figure S68. ^{13}C NMR spectrum of compound **8**

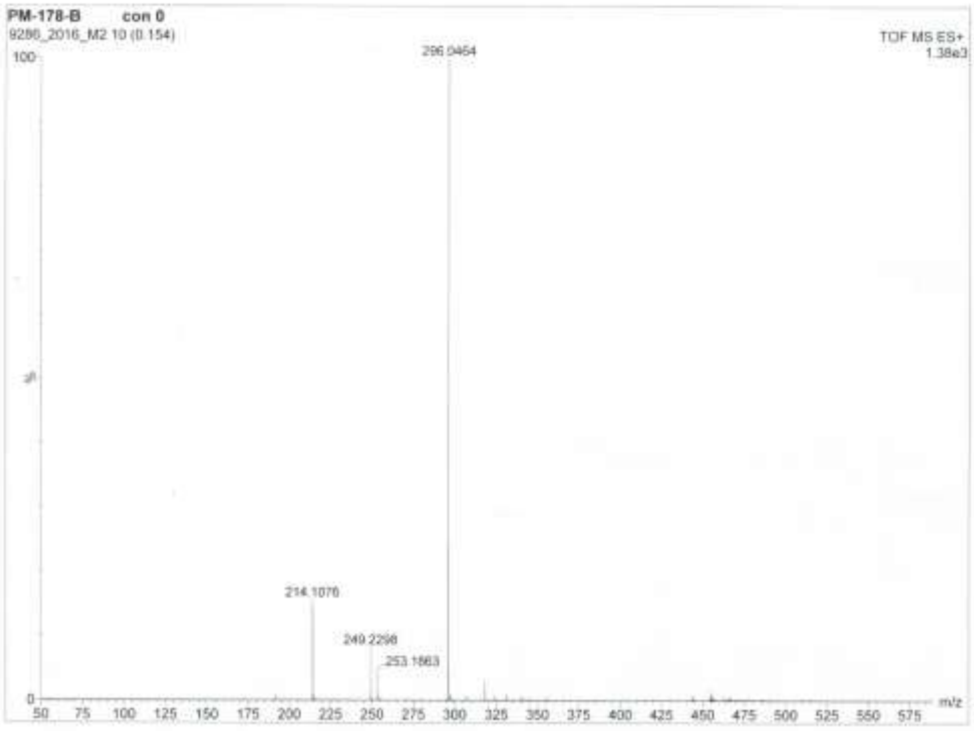

Figure S69. HRMS spectrum of compound **8**

*mixture of compounds **9a** and **10***

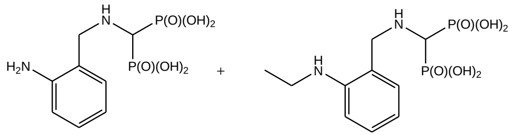

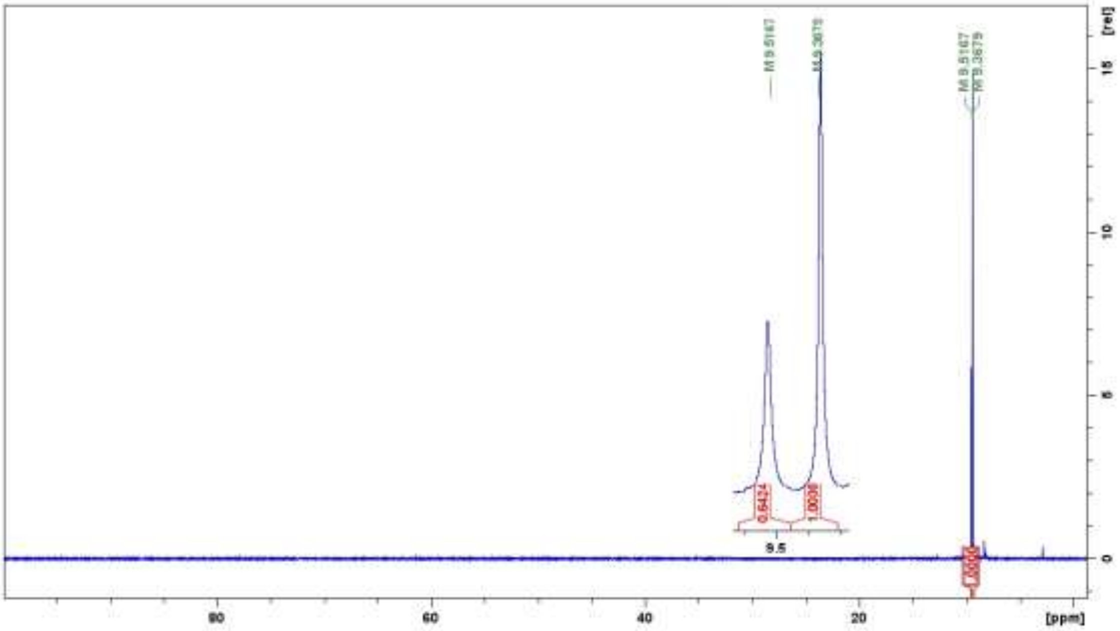

Figure S70. ^{31}P NMR spectrum of mixture of compounds **9a and 10**

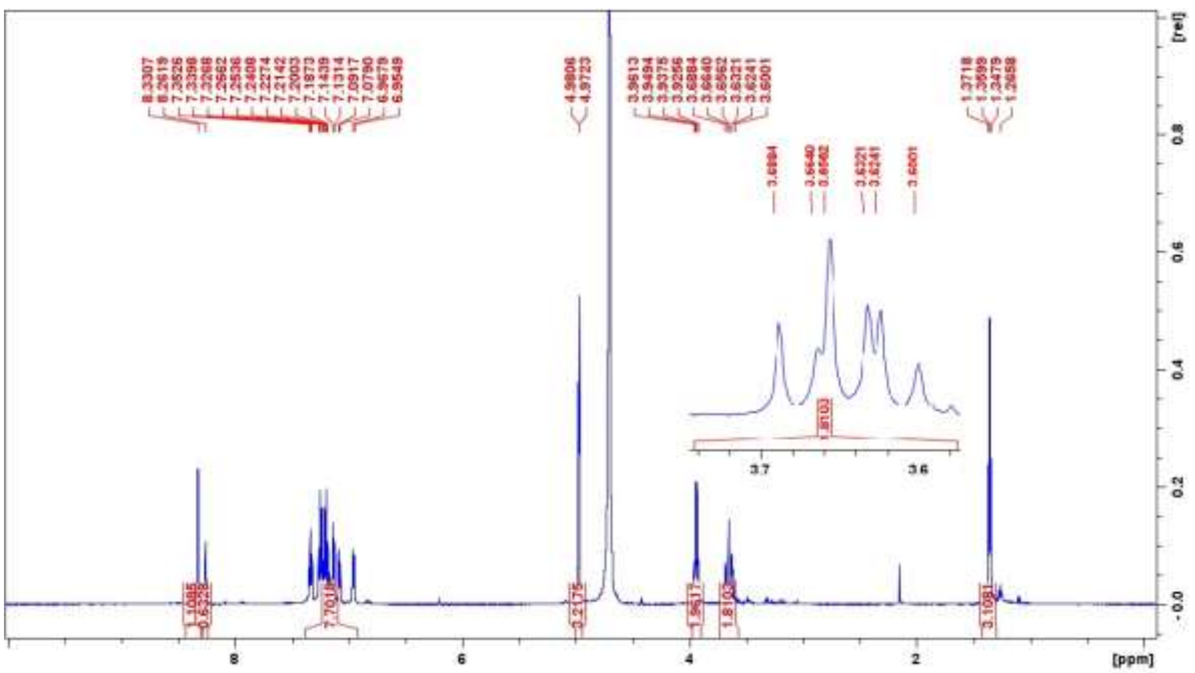

Figure S71. ^{1}H NMR spectrum of mixture of compounds **9a** and **10**

*compound **9b***

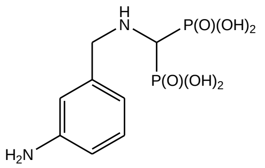

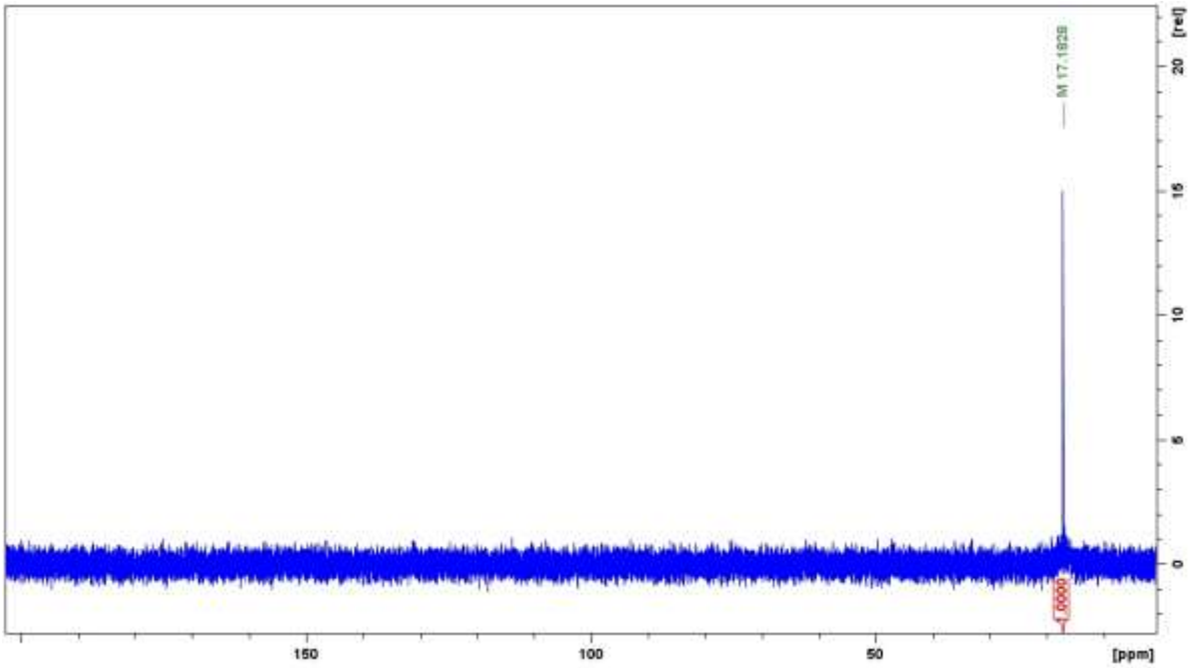

Figure S72. ^{31}P NMR spectrum of mixture of compounds **9b**

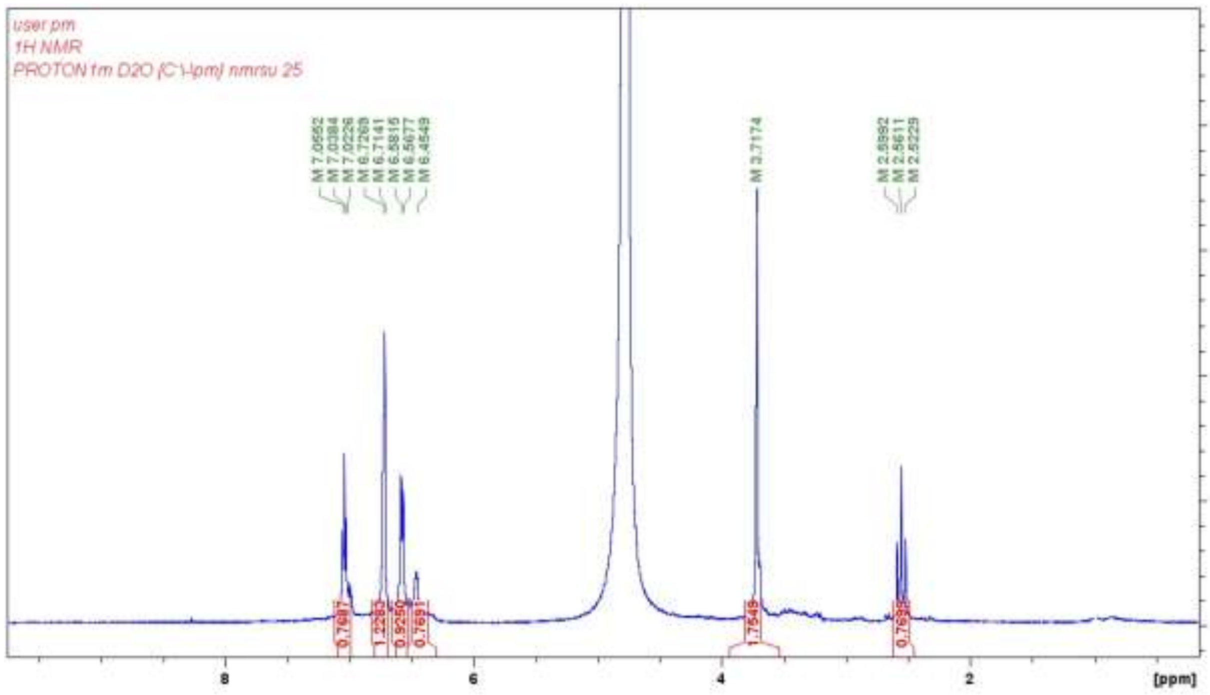

Figure S73. ^{1}H NMR spectrum of compound ***9b***

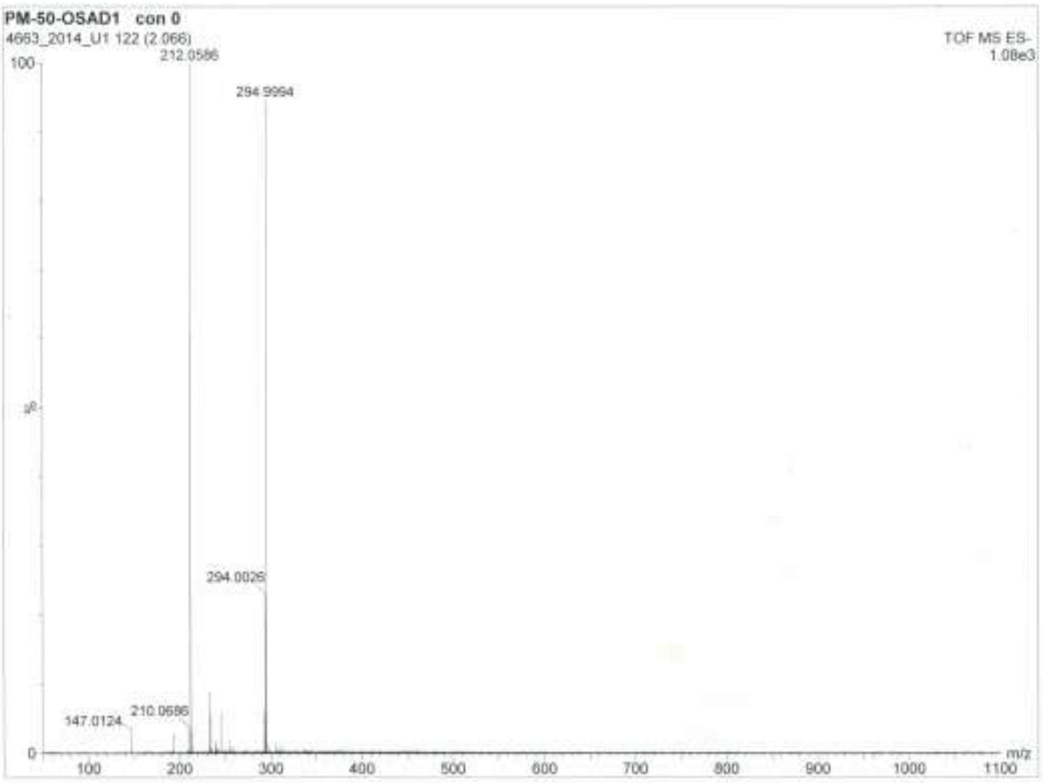

Figure S74. *^{1}H NMR spectrum of compound* ***9b***
